# Anti-Inflammatory Pharmacological Mechanism Mediated by the Conversion of Glycosides to Aglycones in Fangfeng (*Saposhnikoviae Radix*) in Rheumatoid Arthritis Models Based on Serum Metabolomics, Network Pharmacology, and Molecular Docking

**DOI:** 10.3390/ijms26157088

**Published:** 2025-07-23

**Authors:** Wenguang Jing, Xiaoyu Lin, Wenmin Pi, Fangliang He, Haonan Wu, Xianrui Wang, Jia Chen, Xianlong Cheng, Penglong Wang, Feng Wei

**Affiliations:** 1 National Institutes for Food and Drug Control, Beijing 102629, China; jingwenguang@nifdc.org.cn (W.J.);; 2 School of Chinese Pharmacy, Beijing University of Chinese Medicine, Beijing 102488, China

**Keywords:** *Saposhnikoviae Radix*, rheumatoid arthritis, metabolomics, network pharmacology, anti-inflammatory

## Abstract

This study aims to explore the anti-inflammatory pharmacological components and anti-inflammatory mechanisms of the alcohol extract of *Saposhnikoviae Radix* (SR). The components of the alcohol extract of SR were analyzed using the UPLC-MS/MS system. The anti-inflammatory efficacy of the alcohol extract and core components of SR was evaluated using the LPS-induced inflammation model of RAW264.7 cells. The anti-inflammatory mechanism of SR in a mouse model of rheumatoid arthritis was expounded by means of serum metabolomics, network pharmacology, and molecular docking. A total of 12 chromones and 13 coumarins were identified in the alcohol extract of SR. The alcohol extract of SR and its components all had good anti-inflammatory activities. In the mouse model of rheumatoid arthritis, the glycoside compounds of SR were transformed into aglycones, thereby exerting anti-inflammatory effects. Moreover, the alcohol extract of SR alleviated the inflammatory response by up-regulating the expression levels of metabolites such as phenylalanine and tyrosine. Network pharmacology and molecular docking results show that SR could exert an anti-inflammatory effect by regulating AGE-RAGE, PI3K-Akt, TNF, MAPK, and Toll-like signaling pathways. In this study, the anti-inflammatory efficacy and mechanisms of the alcohol extract of SR are explored, with the aim of providing a reference for subsequent research.

## 1. Introduction

Rheumatoid arthritis (RA) is a chronic disease characterized by systemic inflammation and autoimmune reactions, mainly manifested as joint inflammation and bone destruction [1]. It is called “the incurable cancer”, and there is currently no cure for it [2]. RA causes severe injuries to hands and feet, affecting from 0.5% to 1.0% of the global population [3]. With the emergence of new drugs and the accumulation of scientific evidence, the diagnosis and management of rheumatoid arthritis have been constantly updated, and more patients are now able to achieve remission of the disease [4]. Traditional herbal medicines have been used for over a thousand years of clinical diagnosis and treatment experience. Numerous reports have indicated that the extracts, monomers, and active ingredients of some traditional herbal medicines have good anti-rheumatoid arthritis activities [5].

Among them, Fangfeng (*Saposhnikoviae Radix*, SR), which has the function of dispelling wind and dampness, has outstanding performance in the treatment of rheumatoid arthritis [6,7]. SR is derived from the dried roots of *Saposhnikovia divaricata* (Turcz.) Schischk., a plant of the umbelliferae family. SR has the effect of dispelling wind and relieving exterior symptoms [8]. The *Pharmacopoeia of the People’s Republic of China* records that SR can be used for treating the common cold and headache, rheumatic arthralgia, rubella and itching, tetanus, and other diseases. Modern research shows that the core active ingredients in SR include chromones [9], coumarins, fatty acid [10], polysaccharide [11], etc. It has pharmacological activities such as anti-inflammation [12,13], anti-oxidation [14], and so on. Chromones compounds, as the core active components of SR, have significant therapeutic effects in rheumatoid arthritis and could improve the arthritis index, swelling degree, and inflammatory factor levels in model mice [15,16,17]. However, in the current literature on the anti-rheumatoid arthritis effects of SR, there were still relatively few studies on the absorption and metabolism of the active ingredients in model mice.

**Scheme 1:**
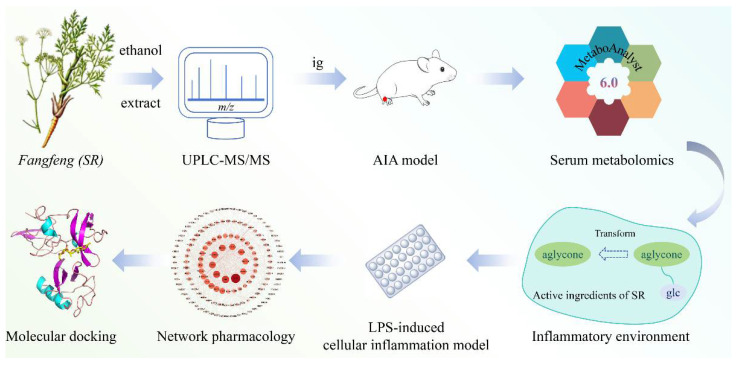
Anti-inflammatory pharmacological mechanism mediated by the conversion of glycosides to aglycones in Fangfeng (*Saposhnikoviae Radix*) based on serum metabolomics, network pharmacology, and molecular docking.

In this study, based on the UPLC-MS/MS technology, a systematic chemical composition analysis of the alcohol extract of SR was conducted. An LPS-induced cellular inflammation model was established to determine the anti-inflammatory effects of the alcohol extract and pharmacological components of SR. A rheumatoid arthritis model in mice was established, and, combined with serum metabolomics, network pharmacology, and molecular docking, the core anti-inflammatory active components and anti-inflammatory mechanisms of SR were analyzed. This study explores the material basis of the anti-inflammatory efficacy of SR, with the aim of providing a reference for subsequent research on SR (Scheme 1).

## 2. Results

### 2.1. Identification of the Constituents in Saposhnikoviae Radix

**Figure 1:**
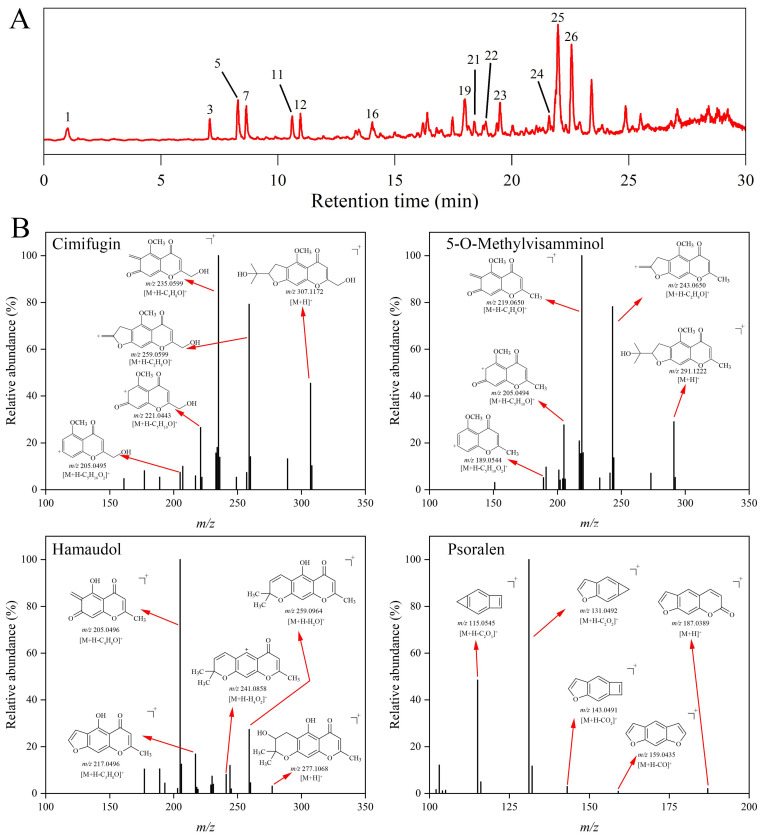
Chemical constituents identified of *Saposhnikoviae Radix*. (**A**) Total ion flow diagram of *Saposhnikoviae Radix* (positive ion mode). (**B**) The mass spectrometry cracking law of cimifugin, 5-O-methylvisamminol, hamaudol, and psoralen.

In this study, a total of 26 compounds are preliminarily identified, including 12 chromones and 13 coumarins (Figure 1A, Table 1). The cracking laws of representative compounds are shown in Figure 1B.

#### 2.1.1. Furanochromone

The most basic structure of chromone was benzo-γ-pyranone, and a dihydrofuran ring was added to its core benzene ring to form furanochromone. The representative compounds of furanochromone in SR were prim-O-glucosylcimifugin and 5-O-methylvisammioside. Compound **3** was identified as prim-O-glucosylcimifugin; its protonated molecule [M+H]^+^ was observed at *m*/*z* 469.1700, followed by the loss of a glucose molecule [M+H-C_6_H_10_O_5_]^+^. Compound **5** was identified as cimifugin, an aglycone of prim-O-glucosylcimifugin, whose precursor ion was observed at *m*/*z* 307.1172. It produced the fragment ion *m*/*z* 289.1070, formed by the loss of H_2_O [M+H-H_2_O]^+^. Then, by continuously losing CH_3_, C_2_H_3_, C_3_H_3_, C_4_H_5_, and C_4_H_5_O molecules, many fragments of cimifugin could be observed, such as *m*/*z* 274.0825 [M+H-CH_5_O]^+^, *m*/*z* 205.0495 [M+H-C_5_H_10_O_2_]^+^, and so on.

Compound **7** was identified as 5-O-methylvisammioside (*m*/*z* 453.1748 [M+H]^+^), which had a cracking law similar to that of prim-O-glucosylcimifugin. It lost one glucose molecule to obtain the aglycone 5-O-Methylvisamminol (compound **11**, *m*/*z* 291.1222 [M+H]^+^). Many fragment ions, such as *m*/*z* 231.0653 [M+H-C_3_H_8_O]^+^ and *m*/*z* 219.0650 [M+H-C_4_H_8_O]^+^, could be obtained by continuous loss of C_2_H_3_, C_3_H_3_, and other fragments from the protonated ion. In conclusion, the cleavage law of furanochromone compounds was as follows: molecular ions were more inclined to lose H_2_O, CH_3_, and other groups in the dihydrofuran ring and its branch chains to obtain fragment ions, and their cracking law is shown in Figure S1.

#### 2.1.2. Pyranochromone

The representative compound of pyranopyranochromogen was sec-O-glucosylhamaudol (compound **12**), and its precursor ions could be observed at *m*/*z* 439.1592 [M+H]^+^. The fragment ion *m*/*z* 277.1068 [M+H-C_6_H_10_O_5_]^+^ obtained after the removal of a molecule of the glucose group from sec-O-glucosylhamaudol was identified as hamaudol (compound **16**, *m*/*z* 277.1067 [M+H]^+^). Hymolol continuously lost two molecules of H_2_O, and fragment ions [M+H-H_2_O]^+^ and [M+H-H_4_O_2_]^+^ could be observed at *m*/*z* 259.0964 and *m*/*z* 241.0858. Fragment ions of hamaudol could also be observed at *m*/*z* 217.0496 due to the loss of H_2_O and C_3_H_6_. Different from furanochromone, hamaudol could produce fragment ions *m*/*z* 205.0496 by losing a C_4_H_8_O fragment through RDA cleavage (Figure S2).

Compound **26** was identified as 3′-O-angeloylhamaudol, distinguished from sec-O-glucosylhamaudol by the 3′ position. Its protonated molecule [M+H]^+^ could be found at *m*/*z* 359.1481, easily losing 3′ groups to obtain fragment ions of *m*/*z* 259.0963 [M+H-C_5_H_8_O_2_]^+^. Subsequently, fragment ions *m*/*z* 241.0856 [M+H-C_5_H_10_O_3_]^+^, *m*/*z* 231.1013 [M+H-C_6_H_8_O_3_]^+^, and *m*/*z* 217.0496 [M+H-C_8_H_14_O_2_]^+^ could also be obtained by losing H_2_O, CO, and C_3_H_6_ molecules. In addition, fragment ions [M+H-C_9_H_14_O]^+^ were also observed at *m*/*z* 205.0495 after RDA cleavage.

#### 2.1.3. Coumarin

Coumarins are benzoine-α-pyranones which most often appear in mass spectrometry as a series of CO loss fragment ion peaks (Figure S3). Compound **14** was identified as psoralen, whose molecular ion peak could be observed in *m*/*z* 187.0389, and by continuous loss of CO and CO_2_, fragment ions could be observed in *m*/*z* 159.0435 [M+H-CO]^+^, *m*/*z* 143.0491 [M+H-CO_2_]^+^, *m*/*z* 131.0492 [M+H-C_2_O_2_]^+^, and *m*/*z* 115.0545 [M+H-C_2_O_3_]^+^.

Substituting coumarins were also prone to a loss of substituents. For example, compound 20 (*m*/*z* 271.0964 [M+H]^+^) was identified as imperatorin, and its fragment ions were observed at *m*/*z* 203.0339, formed by the loss of C_5_H_8_ fragments [M+H-C_5_H_8_]^+^. On this basis, two molecules of CO could be lost continuously to obtain fragment ions *m*/*z* 175.0390 [M+H-C_6_H_8_O]^+^ and *m*/*z* 147.0441 [M+H-C_7_H_8_O_2_]^+^. Compound **24** was identified as osthenol (*m*/*z* 231.1012 [M+H]^+^), and its fragmentation pattern was similar to that of imperatorin, which first lost the substituent of the side chain (*m*/*z* 163.0389 [M+H-C_5_H_8_]^+^), and then lost two molecules of CO successively to obtain its fragment ion (*m*/*z* 135.0439 [M+H-C_6_H_8_O]^+^, *m*/*z* 107.0495 [M+H-C_7_H_8_O_2_]^+^).

### 2.2. Identification of Metabolites In Vivo of Saposhnikoviae Radix

#### 2.2.1. Exogenous Metabolites Analysis

**Figure 2:**
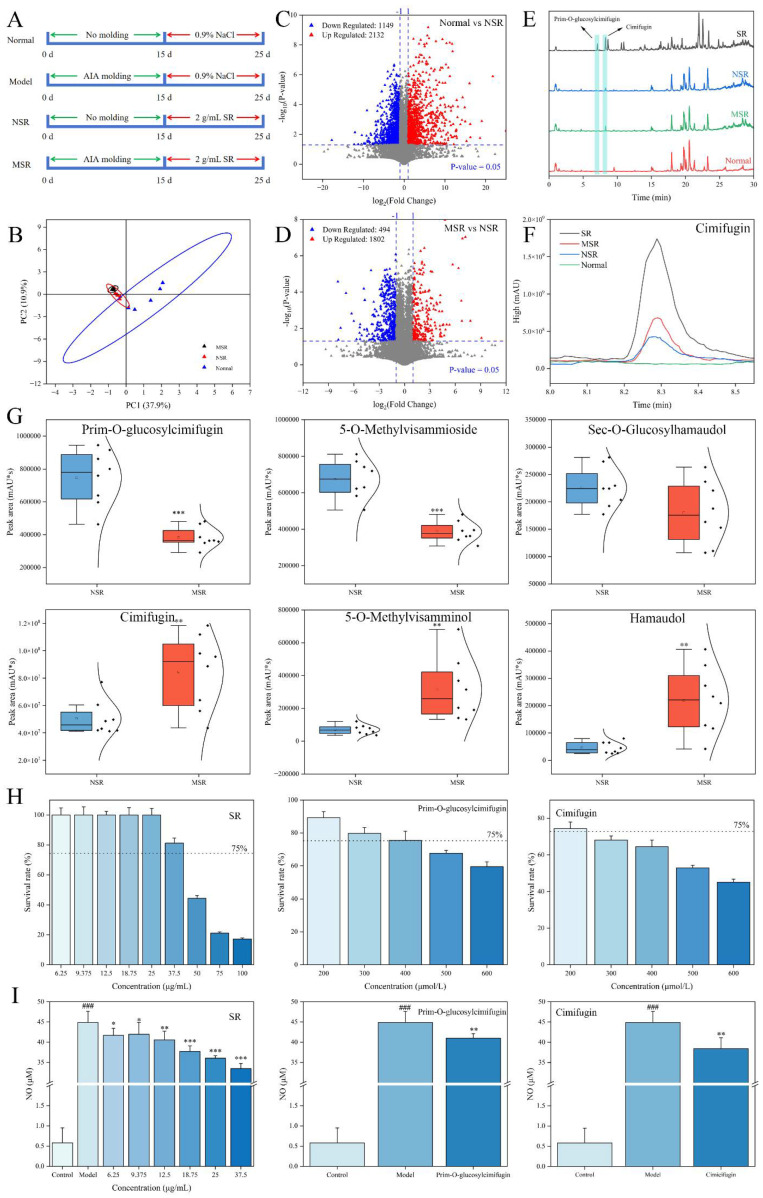
Analysis of exogenous metabolites of *Saposhnikoviae Radix*. (**A**) Experimental scheme. (**B**) PCA. (**C**) Volcano plot of normal group and NSR group. (**D**) Volcano plot of MSR group and NSR group. (**E**) Total ion flow diagram of each group of samples. (**F**) Peak area of cimifugin in each group of samples. (**G**) The content of exogenous components in the NSR group and MSR group. ** *p* < 0.01, *** *p* < 0.001, compared to the NSR group. (**H**) Toxicity of the samples to RAW264.7. (**I**) NO content of each sample. ^###^ *p* < 0.001, compared to the control group. * *p* < 0.05, ** *p* < 0.01, *** *p* < 0.001, compared to the model group.

The administration scheme of each group of mice is shown in Figure 2A. Through analysis of the normal group, NSR group, and MSR group, the exogenous blood entry components of SR were identified, and the differences in exogenous blood entry components between normal mice and model mice were compared.

A principal component analysis (PCA) showed that the normal group, MSR group, and NSR group could be significantly separated, indicating obvious differences among the three groups (Figure 2B). The MSR group and NSR group were close, indicating that the metabolites of mice given SR was obviously different from that of normal mice. A volcano plot shows that there were 1149 down-regulated metabolites and 2132 up-regulated metabolites in the NSR group compared to the normal group (Figure 2C). Furthermore, the endogenous metabolites shared by the normal group and NSR group were excluded to screen the significantly different entry prototype components. A total of eight prototypic components were identified, including divaricatacid, prim-O-glucosylcimifuginm, cimifugin, 5-O-methylvisammioside, norcimifugin, 5-O-methylvisammioside 5-O-methylvisamminol, sec-O-glucosylhamaudol, and hamaudol (Table 2). The differences between the NSR group and SR group were further analyzed and compared, and the metabolites of SR entering the blood were identified. A total of six metabolites were identified, and these metabolites mainly underwent phase I metabolic reactions, such as oxidation, demethylation, and desaturation, and phase II metabolic reactions, such as glucuronide conjugation, in mice (Table 2).

M1 and M2 have been identified as oxidation products of cimifugin, and are isomers of each other, and their excimer ions [M+H]^+^ could be observed at *m*/*z* 323.1122 and *m*/*z* 323.1124. The relative molecular weight of the fragment ions was 16 (O), more than that of cimifugin (M7), and the cracking law of the two fragments was basically the same as that of cimifugin. For example, the characteristic fragment ions of M1 were *m*/*z* 305.1019 [M+H-H_2_O]^+^, *m*/*z* 259.0604 [M+H-C_2_H_8_O_2_]^+^, *m*/*z* 247.0602 [M+H-C_3_H_8_O_2_]^+^, *m*/*z* 235.0600 [M+H-C_4_H_8_O_2_]^+^, and *m*/*z* 221.0442 [M+H-C_5_H_10_O_2_]^+^. M8 has been identified as a desaturation product of cimifugin and its excimer ion [M+H]^+^ could be observed at *m*/*z* 305.1020. The relative molecular mass of the fragment ions was 2 (2H) more than that of cimifugin (M7). The characteristic fragment ions were *m*/*z* 289.1065 [M+H-O]^+^, *m*/*z* 274.0829 [M+H-CH_3_O]^+^, *m*/*z* 259.0598 [M+H-C_2_H_6_O]^+^, *m*/*z* 247.0596 [M+H-C_3_H_6_O]^+^, *m*/*z* 235.0598 [M+H-C_4_H_6_O]^+^, *m*/*z* 221.0442 [M+H-C_5_H_8_O]^+^, and *m*/*z* 205.0493 [M+H-C_5_H_8_O_2_]^+^.

The contents of prim-O-glucosylcimifuginm and cimifugin in the MSR group and NSR group were different according to the total ion flow diagram (Figure 2E,F). The three groups of representative glycosides and aglycones were selected for semi-quantitative analysis; the results show that the content of glycosides (prim-O-glucosylcimifuginm, 5-O-methylvisammioside, and sec-O-glucosylhamaudol) in the NSR group were generally higher than that in the MSR group, while the corresponding aglycones (cimifugin, 5-O-methylvisamminol, and hamaudol) in the MSR group were significantly higher than those in the NSR group (Figure 2G). We established a model of LPS-induced cellular inflammation for further verification (Figure 2H,I). SR had no significant cytotoxic effect in the range of 6.25–37.5 μg/mL. In this range, SR could significantly inhibit the production of NO, indicating that SR had excellent anti-inflammatory activity. Both prim-O-glucosylcimifuginm and cimifugin could reduce the expression of NO at a dosage of 200 μmol/L, and the anti-inflammatory activity of cimifugin was better than that of prim-O-glucosylcimifuginm. These results suggest that, in rheumatoid arthritis mice, glycosides of SR were more likely to break bonds into aglycones and play an anti-inflammatory role. It is speculated that the physiological environment of rheumatoid arthritis mice is different from that of normal mice, which affects the metabolic transformation process of pharmacodynamic components.

#### 2.2.2. Endogenous Metabolites Analysis

**Figure 3:**
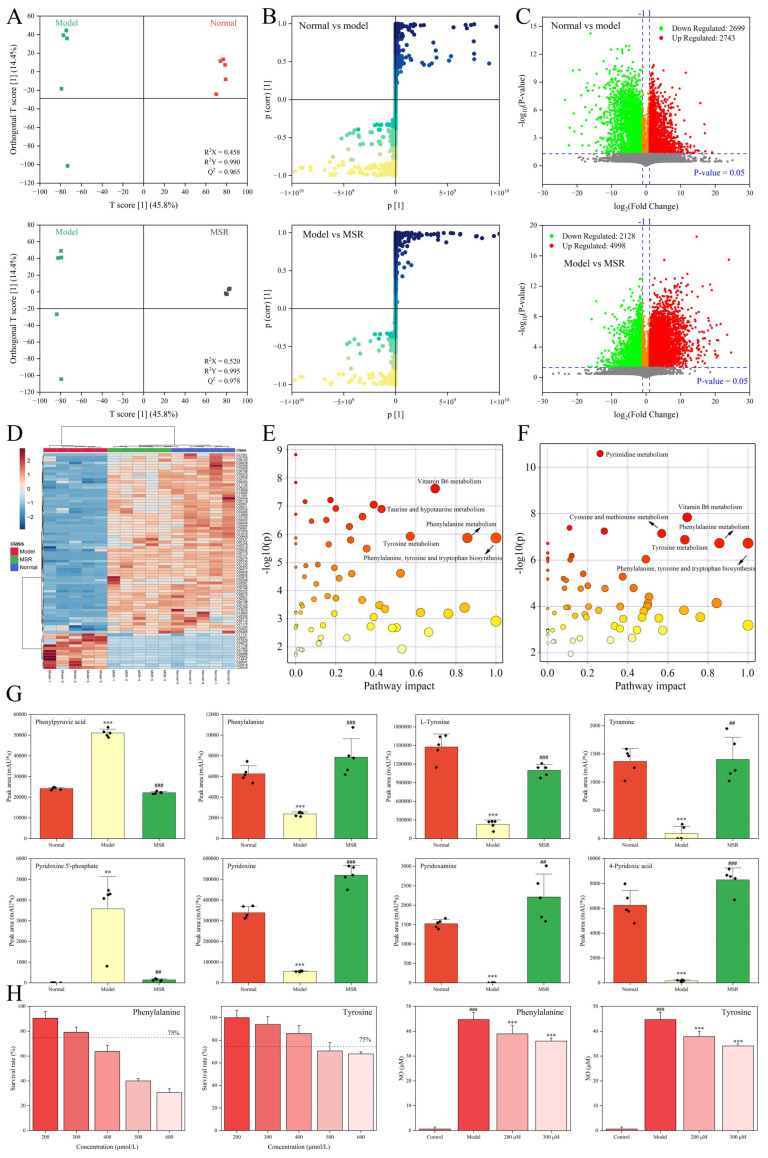
Analysis of endogenous metabolites of *Saposhnikoviae Radix*. (**A**) OPLS-DA analysis. (**B**) S-plot analysis. (**C**) Volcano plot. (**D**) Clustering heat map (top 25%). (**E**) Metabolic pathway of normal vs. model. (**F**) Metabolic pathway of model vs. MSR. (**G**) The content of differences endogenous metabolite in each group. ** *p* < 0.01, *** *p* < 0.001, compared to the normal group. ^##^ *p* < 0.01, ^###^ *p* < 0.001, compared to the model group. (**H**) LPS induced cell inflammatory model. ^###^ *p* < 0.001, compared to the control group. *** *p* < 0.001, compared with model group.

Serum metabolites in the normal group, model group, and MSR group were analyzed using non-targeted metabolomics to explain the pharmacodynamic material basis of SR. An orthogonal partial least squares discriminant analysis (OPLS-DA) showed that the normal group, model group, and MSR group could be significantly separated, suggesting that the intervention of SR could significantly change the metabolic profile of AIA mice (Figure 3A). Moreover, there was no overfitting phenomenon in their model, and the values of R2X, R2Y, and Q2 were all suitable. Furthermore, the S-plot of OPLA-DA analysis suggests that the closer to the lower right corner and upper left corner, the more significant the difference (Figure 3B). With variable importance in projection (VIP) > 1 as the screening condition, a total of 6308 endogenous differential metabolites were screened in the normal vs. model groups, and 7102 endogenous differential metabolites were screened in the model vs. MSR groups. By marking the up-down-regulation of metabolites in each group in green and red, the results show that, compared to the normal group, 2699 endogenous metabolites in the model group were down-regulated and 2743 endogenous metabolites were up-regulated, while in the model vs. MSR, a total of 2128 endogenous metabolites were down-regulated and 4998 endogenous metabolites were up-regulated (Figure 3C).

Under the conditions of “*p* < 0.05”, “VIP > 1”, and “Fold change < 0.5 or Fold change > 2”, potential endogenous differential metabolites were screened. There were 316 different metabolites found in the normal vs. model groups, and 350 different metabolites found in the model vs. MSR groups, among which 231 were common endogenous different metabolites (Table S1). The expression level of endogenous metabolites in the normal group and MSR group was more similar, which was significantly different from that in the model group, indicating that the metabolites of AIA mice had a tendency to return to a normal state after SR treatment (Figure 3D).

A metabolic pathway enrichment map of mouse serum was constructed using MetaboAnalyst 5.0, and the metabolic pathways meeting the conditions of “pathway impact > 0” and “−log (*p*) > 0.5” were screened. It was found that the effect of SR on AIA mice was related to vitamin B6 metabolism, phenylalanine, tyrosine and tryptophan biosynthesis, phenylalanine metabolism, tyrosine metabolism, and other metabolic pathways (Figure 3E,F). Studies have shown that there was a significant accumulation of phenylpyruvic acid in diabetic foot ulcers, and the increase in phenylpyruvic acid could cause the imbalance of macrophage polarization and enhance the inflammatory response, which damaged wound healing [18]. The expression level of phenylpyruvic acid in the model group was up-regulated, while that in the MSR group it was down-regulated, suggesting that the inflammatory response in the MSR group was alleviated (Figure 3G). Phenylalanine inhibited the production of IL-1β and TNF-α in pro-inflammatory (M1) macrophages, and supplementation with phenylalanine attenuated macrophage-mediated inflammation in vivo [19]. Vitamin B6 is a general term for pyridoxine, pyridoxamine, and its phosphorylated derivatives, which could reduce type 2 inflammation by regulating IL-33 homeostasis [20]. Except for phenylpyruvic acid, the expression levels of phenylalanine, L-tyrosine, tyramine, pyridoxine, pyridoxamine, and 4-pyridoxic acid in the serum of the model group were significantly lower than those in the normal group (*p* < 0.001), while the expression level of pyridoxine 5′-phosphate was significantly higher than that of the normal group (Figure 3G). The above changes were reversed after SR intervention, and the expression level of metabolites in the MSR group was gradually inclined to that in the normal group. The metabolites phenylalanine and tyrosine also had good anti-inflammatory activity at 200 μmol/L and 300 μmol/L (Figure 3H). Combining the exogenous metabolism and endogenous metabolism, it is speculated that SR could further enhance the expression level of phenylalanine, tyrosine, and other metabolites by promoting the transformation of core pharmacodynamic components, such as prim-O-glucosylcimifuginm into aglycone (cimifuginm), and synergistically perform anti-inflammatory activities.

### 2.3. Anti-Inflammatory Mechanism of Saposhnikoviae Radix

#### 2.3.1. Network Pharmacology Analysis

**Figure 4:**
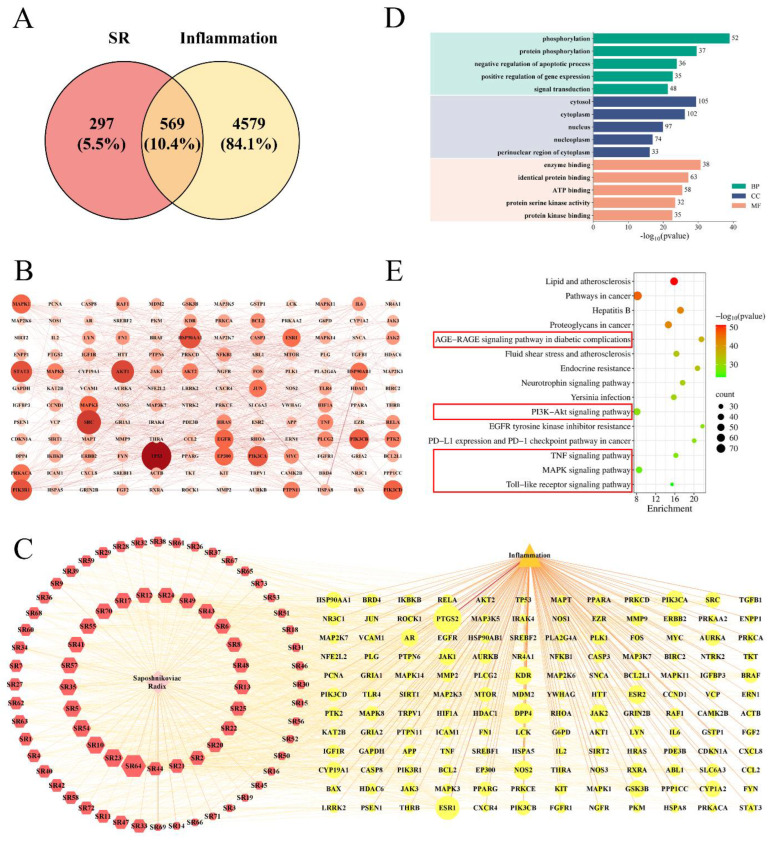
Network pharmacology analysis of *Saposhnikoviae Radix*. (**A**) Venn diagram of *Saposhnikoviae Radix*-inflammatory targets. (**B**) PPI network of potential anti-inflammatory targets of *Saposhnikoviae Radix* (the size and color intensity of the nodes represent the magnitude of the degree value). (**C**) Network diagram of *Saposhnikoviae Radix*-components-targets-inflammation (pink represents herb, red represents active ingredients, orange represents diseases, yellow represents targets, and node size represents degree values). (**D**) GO enrichment analysis. (**E**) KEGG enrichment analysis. The red boxes represented the pathways related to inflammation.

We obtained 73 active ingredients and 866 targets of SR, and 5148 targets that were inflammatory. After taking the intersection, we obtained 569 common targets between SR and inflammation, as shown in Figure 4A. The common targets were subjected to a PPI network analysis, which included 565 nodes and 1931 edges, with an average node connectivity of 6.84. Using a topological analysis, 144 core targets were identified, including TP53, SRC, AKT1, STAT3, JUN, HSP90AA1, and so on (Figure 4B, Table S2). Additionally, the “herb-component-target-disease” network diagram was constructed using the core targets, which included 219 nodes and 1209 edges, as shown in Figure 4C and Table S3. Among the active ingredients of SR, in addition to the blood entry components Divaricatacid (SR58), Prim-O-glucosylcimifuginm, Cimifugin (SR12), 5-O-methylvisammioside (SR71), Norcimifugin (SR72), 5-O-methylvisamminol (SR18), Sec-O-Glucosylhamaudol (SR73), and Hamaudol, compounds such as sitosterol (SR64) and 5-[(Z)-2-(4-hydroxy-3-methoxy-phenyl)vinyl]resorcinol (SR23) also had high degree values, and thus were collectively used as core components for subsequent molecular docking validation. Furthermore, GO and KEGG enrichment analyses were conducted using the core targets. A total of 772 BPs (biological processes), 114 CCs (cellular components), and 151 MFs (molecular functions) were obtained with a screening criterion of *p* < 0.05. The top five entries ranked by *p*-value were selected for inclusion in the GO enrichment analysis bar charts. As shown in Figure 4D, this includes protein phosphorylation, regulation of apoptosis, regulation of gene expression, signal transduction, and so on, primarily through inhibiting apoptosis and protecting cells to achieve an anti-inflammatory effect. A total of 177 pathways were obtained with a screening criterion of *p* < 0.05, and the 15 pathways were selected for plotting, as shown in Figure 4E. The AGE-RAGE, PI3K-Akt, TNF, MAPK, and Toll-like signaling pathways are all closely associated with inflammation. Therefore, SR may exert its anti-inflammatory effect by inhibiting the AGE-RAGE, PI3K-Akt, TNF, MAPK, and Toll-like signaling pathways.

#### 2.3.2. Molecular Docking Verification

**Figure 5:**
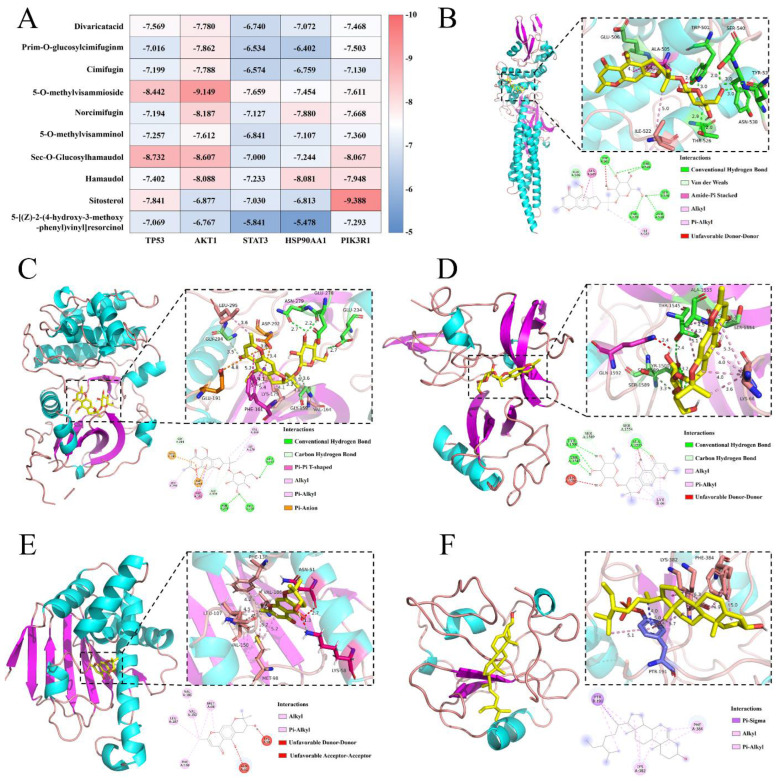
Molecular docking of *Saposhnikoviae Radix. (***A**) Heatmap of molecular docking scores for *Saposhnikoviae Radix* anti-inflammatory effects. (**B**–**F**) Molecular docking results of STAT3 and 5-O-methylvisammioside, TP53 and Sec-O-Glucosylhamaudol, AKT1 and 5-O-methylvisammioside, HSP90AA1 and Hamaudol, and PIK3R1 and Sitosterol.

Combining PPI network analysis and KEGG enrichment analysis, we found that the top ranked core targets in the PPI network (TP53, AKT1, STAT3, JUN, and HSP90AA1) were also key targets in AGE-RAGE, PI3K-Akt, TNF, MAPK, and Toll-like signaling pathways. Therefore, these five core targets performed molecular docking with the core components (Divaricatacid, Prim-O-glucylcimifugin, Cimifugin, 5-O-methylvisammioside, Norcimifugin, 5-O-methylvisamminol, Sec-O-Glucylhamaudol, Hamaudol, sitosterol, and 5-[(Z)-2-(4-hydroxy-3-methoxy-phenyl)vinyl] resorcinol). The obtained data was analyzed using a heatmap, and the results are shown in Figure 5A. The conformation with the highest score for each target protein was analyzed by plotting, as shown in Figure 5B–F. Among them, there were 39 groups with docking scores ≤ −7.0 kcal/mol, accounting for 78%. It is generally believed that a docking score ≤ −5.0 kcal/mol indicates good bonding strength between ligand and receptor, and a docking score ≤ −7.0 kcal/mol indicates stronger bonding strength. From the composition perspective, 5-O-methylvisamminol, Sec-O-Glucylhamaudol, and Hamaudol bound strongly to each target protein; from the target protein’s perspective, AKT1 (AKT serine/threonine kinase 1) and PIK3R1 (Phosphoinositide-3-Kinase Regulatory Subunit 1) had a strong binding affinity with various active ingredients. The binding mode between the active ingredients and the target proteins were mainly hydrogen bonding and hydrophobic interaction. Therefore, the main active ingredients in the SR that entered the blood had strong binding abilities with target proteins in AGE-RAGE, PI3K-Akt, TNF, MAPK, and Toll-like signaling pathways (Table S4).

## 3. Discussion

An increasing body of research is showing that the pathogenesis of RA is diverse and still needs to be further explored [21]. Under the combined influence of multiple factors such as genetics, infection, and the environment, the immune damage and repair caused by autoimmune reactions are the basis for the occurrence and development of rheumatoid arthritis. And manifests as abnormal increases in T cells, B cells, macrophages, inflammatory cytokines, chemokines, and autoantibodies [22]. In this study, regarding the pharmacological efficacy evaluation of Saposhnikoviae Radix for rheumatoid arthritis, the absorption of the active ingredients in the blood, from the perspective of inflammation, was analyzed. However, the mechanism of immune treatment for rheumatoid arthritis through Saposhnikoviae Radix still required further exploration.

Gut microbiota imbalance has been proven to be closely related to the pathogenesis of rheumatoid arthritis. Parabacteroides distasonis is a probiotic used to treat rheumatoid arthritis. Oral administration of Parabacteroides distasonis in mice with rheumatoid arthritis could significantly improve their symptoms [23]. During the process of converting the pharmacological components in Saposhnikoviae Radix from glycosides to aglycones, it was further speculated that not only were enzymes involved, but also that the intestinal flora played a crucial role, working together to exert the therapeutic effect. Therefore, the intestinal microbiota could also serve as a very effective starting point to explain the pharmacological mechanism of Saposhnikoviae Radix in treating rheumatoid arthritis.

## 4. Materials and Methods

### 4.1. Materials and Reagents

SR was collected in Zhanjiang, Guangdong Province, which was identified as genuine by Professor Wei Feng (National Institutes for Food and Drug Control); RAW264.7 (mouse monocyte–macrophage leukemia cells) was purchased from the Cell Bank of Shanghai Chinese Academy of Sciences.

Methyl thiazolyl tetrazolium (MTT), lipopolysaccharide (LPS), and nitric oxide (NO) detection kits were purchased from Beijing Biorigin Biotechnology Co., Ltd. (Beijing, China); Dimethyl sulfoxide (DMSO) was purchased from Beijing Chemical Plant (Beijing, China); Methanol (analytical pure) and 95% ethanol (analytical pure) were purchased from Sinopharm Chemical Reagent Company (Beijing, China); and the water was Merk Millipore purified water (Billerica, MA, USA).

### 4.2. Sample Preparation

The SR was crushed into powder and sifted. Eight times the amount of ethanol (95%) was added into the medicinal powder. The ethanol extract of SR was obtained by boiling it twice with condensation reflux (2 h each time). Then the sample was concentrated under reduced pressure for use.

### 4.3. Systematic Chemical Constituents Analysis of Saposhnikoviae Radix

The sample under item 2.2 was redissolved with methanol to 1 mg/mL, and then the sample to be tested was filtered using a 0.22 μm microporous filtration membrane. TC-C18 (4.6 mm × 250 mm, 5 μm); Acetonitrile (A)−0.1% formic acid solution (B). Elution condition: 0–30 min 2%A~98%A. Flow rate: 0.3 mL/min; column temperature: 35 °C; injection volume: 1 μL. Information was collected in positive spray ionization mode (ESI+). Both the capillary temperature and the auxiliary gas heater were set to 350 °C. The scanning mode was Full MS, the resolution was 10,000, and the scanning range was *m*/*z* 150–1500.

### 4.4. Analysis of Metabolites In Vivo of Saposhnikoviae Radix

After 7 days of adaptive feeding, C57 male mice were randomly divided into four groups: the normal group, the model group, the NSR group, and the MSR group. The mice in the model group and MSR group were modeled for 15 days (Adjuvant induced rheumatoid arthritis: AIA), and the mice in the normal group and NSR group were not modeled. After 15 days, the NSR group and MSR group were given an alcohol extract of SR; the dosage was 2 g/mL (calculated by raw drug) and the administration volume was 0.1 mL/10 g. The normal group and model group were given the same amount of normal saline. After 10 days of continuous administration, the plasma of each group of mice was collected, and a heparin sodium tube was used.

The plasma of the mice in each group was centrifuged at 3000 rpm/min at 4 °C for 15 min, and the supernatant parts (serum) were collected. Then anhydrous methanol was added to a methanol concentration of 75% for protein deposition. The sample was centrifuged at 4 °C at 12,000 rpm/min for 5 min, and the supernatant was taken for nitrogen blowing treatment. The precipitate obtained after nitrogen blowing was redissolved in 75% methanol water, and the metabolomics samples were obtained using a 0.22 μm microporous filtration membrane.

### 4.5. Analysis of Exogenous Components

Identification of exogenous components: Serum mass spectrometry of the normal group and NSR group was compared using Progenesis software 2.0 to exclude the interference of endogenous components. The remaining components were compared using the molecular weight, retention time, and secondary mass spectrum fragment information of the SR group. If the two results were consistent, it was considered to be a prototype component of SR entering blood. There were no components in the mass spectra of the normal group and SR group, and only the components in the NSR group were considered to be metabolites of SR.

### 4.6. Analysis of Endogenous Components

After deducting exogenous metabolites, “*p* < 0.05”, “VIP > 1”, and “Fold change < 0.5 or Fold change > 2” were used as the conditions for screening different metabolites in blood between the normal group, model group, and MSR group. MetaboAnalyst 5.0 (https://www.metaboanalyst.ca/, accessed on 5 September 2024) was used to analyze the metabolic pathways of the selected differential metabolites.

### 4.7. LPS-Induced Inflammation Model of RAW264.7 Cells

RAW264.7 cells were cultured in DMEM high-glucose medium (37 °C, 5% CO_2_). Cells in the logarithmic growth phase were seeded in 96-well plates at a density of 1 × 10^5^ cells/mL. They were then cultured in a constant temperature biochemical incubator for 24 h. The cell safety of SR was screened at a dose of 6.25–100 mg/mL, and the cell safety of prim-O-glucosylcimifugin and cimifugin were screened at a dose of 200–600 μmol/L. After the sample was added to the culture medium to prepare the required concentration, it was transferred to a 96-well plate (100 μL per well) and continued to be cultured in a constant-temperature biochemical incubator for 24 h. Then 5 mg/mL MTT solution was added to the 96-well plate. After incubation at 37 °C in the dark for 4 h, the supernatant was discarded. In total, 150 μL of dimethyl sulfoxide was added to each well. The absorbance was detected at 490 nm, and the cell survival rate was calculated.

The LPS-induced inflammation model of RAW264.7 cells was established, and the NO content among the samples was determined. Cells in the logarithmic growth phase were seeded in 96-well plates at a density of 1 × 10^6^ cells/mL. They were then cultured in a constant-temperature biochemical incubator for 24 h. Based on the MTT evaluation results, the drug suspension was prepared and added to a 96-well plate, with 50 μL per well. After being incubated in a constant-temperature biochemical incubator for 0.5 h, 50 μL of medium containing 1 μg/mL LPS was added to each well. The 96-well plates were placed in a constant-temperature biochemical incubator, and continued to be cultured for 24 h. Subsequently, 50 μL of the cell culture supernatant was taken, and the NO levels of each sample were determined using a nitric oxide (NO) detection kits were purchased from Beijing Biorigin Biotechnology Co., Ltd. (Beijing, China) NO kit.

### 4.8. Network Pharmacology Analysis

Search for the chemical components of SR through the TCMSP database, using OB (oral bioavailability) ≥ 30% and DL (drug-like properties) ≥ 0.18 as screening criteria. Supplement its chemical components by combining previous mass spectrometry analysis results and a literature review. Using the PubChem database to collect the molecular structures of compounds and save them uniformly in the “sdf” format. Use the Swiss Target Prediction and TCMSP databases to predict the targets of the collected active components. Import the obtained “sdf” format file into the Swiss database, using “Homo sapiens” as the research species, and obtain the action targets of active components under the condition of “probability > 0”. Integrate and deduplicate these targets with those obtained from the TCMSP database to obtain the potential action targets of SR. Then use the UniProt database to unify the targets. Utilize the GeneCards database to search for disease targets related to inflammation using the keyword “inflammation”, and filter based on “Relevance score ≥ 1”. Take the intersection of the SR active component targets and the inflammation-related disease targets to obtain the intersection targets, which are the potential action targets. Input the potential targets into STRING to construct a protein–protein interaction network, with the species limited to Homo sapiens and the confidence score set to above 0.9. Hide the disconnected nodes in the network and export the TSV file; then import it into Cytoscape 3.8.0 for visualization to draw the PPI network. Perform a topological analysis and screen the core targets based on three metrics, BC (Betweenness Centrality), CC (Closeness Centrality), and Degree, using the median values of BC, CC, and Degree as the screening criteria. The active ingredients and core targets of SR should then be imported into Cytoscape 3.8.0 software to construct a “herb-ingredient-target-disease” network diagram. The Analyze Network function should be used to analyze the active ingredients of SR. The DAVID website should be utilized to perform GO (Gene Ontology) and KEGG (Kyoto Encyclopedia of Genes and Genomes) enrichment analyses based on the obtained core targets. The identifier should be set to the official gene symbol, and the list type should be set to the gene list, with the species limited to Homo sapiens. The results should be screened with *p* < 0.05.

### 4.9. Molecular Docking

Based on the analysis of blood entry components and the results of “herb-component-target-disease”, 10 core components were selected for molecular docking, including the following: Divaricatacid, Prim-O-glucylcimifugin, Cimifugin, 5-O-methylvisammioside, Norcimifugin, 5-O-methylvisamminol, Sec-O-Glucylhamaudol, Hamaudol, sitosterol, and 5-[(Z)-2-(4-hydroxy-3-methoxy-phenyl)vinyl] resorcinol. The structures of the core components were downloaded from the TCMSP database and saved in mol2 format. Based on the results of the PPI network analysis and pathway enrichment analysis, relevant core targets in the AGE-RAGE, PI3K-Akt, TNF, MAPK, and Toll-like signaling pathways were selected for molecular docking. The structural files of the target proteins were downloaded from the PDB database, including TP53 (PDB ID: 8SVI), AKT1 (PDB ID: 4EKL), STAT3 (PDB ID: 6NJS), HSP90AA1 (PDB ID: 3T0H), and PIK3R1 (PDB ID: 5GJI), using AutoDockTools 1.5.7 to add hydrogen atoms, calculate charges, and save as pdbqt format. We set the docking site and performed molecular docking using AutoDock Vina 1.2.7. Based on the scoring results, we evaluated the binding affinity between each target protein and the small-molecule compounds. We used Pymol 2.3.0 to graphically analyze the high-scoring conformations.

### 4.10. Statistical Analysis

In this study, SPSS software version 20.0 (Armonk, New York, NY, USA) was used to conduct a one-way analysis of variance. Student’s-test was used for statistical significance (*p* < 0.05). All measures were repeated at least three times and are expressed as mean ± standard deviation (X ± SD).

## 5. Conclusions

In this study, with the aid of serum metabolomics, network pharmacology, and molecular docking, the material basis of the anti-inflammatory efficacy of SR was expounded. The core active ingredients of the alcohol extract of SR were chromones and coumarins. The alcohol extract of SR, prim-O-glucosylcimifuginm, and cimifugin all had good anti-inflammatory effects. In mouse models of rheumatoid arthritis, glycosides of SR were converted into aglycones, regulating the expression levels of endogenous metabolites such as phenylalanine and tyrosine, and inhibited the AGE-RAGE, PI3K-Akt, TNF, MAPK, and Toll-like signaling pathways to synergistically exert anti-inflammatory effects.

## Figures and Tables

**Table 1:** Chemical constituents identified from *Saposhnikoviae Radix*.

NO	tR (min)	Compound	Formula	Identity	Theoretical (*m*/*z*)	Experimental (*m*/*z*)	Δppm	Fragment Ion (*m*/*z*)	Classification
1	1.03	Adenosine	C_10_H_13_N_5_O_4_	[M+H]^+^	268.1040	268.1038	−0.75	136.0618 [M+H-C_5_H_8_O_4_]^+^	Other
2	6.96	Divaricatacid	C_16_H_16_O_7_	[M+H]^+^	321.0968	321.0966	−0.62	303.0854 [M+H-H_2_O]^+^ 277.1068 [M+H-CO_2_]^+^ 273.0391 [M+H-C_2_H_8_O]^+^ 249.0390 [M+H-C_3_H_5_O]^+^ 235.0235 [M+H-C_4_H_7_O]^+^	Chromone
3	7.09	Prim-O-glucosylcimifugin	C_22_H_28_O_11_	[M+H]^+^	469.1704	469.1700	−0.85	307.1173 [M+H-C_6_H_10_O_5_]^+^ 289.1068 [M+H-C_6_H_12_O_6_]^+^	Chromone
4	8.05	Scopoletin	C_10_H_8_O_4_	[M+H]^+^	193.0495	193.0495	0.00	178.0258 [M+H-CH_3_]^+^ 150.0314 [M+H-C_2_H_3_O]^+^ 133.0285 [M+H-C_2_H_4_O_2_]^+^ 122.0364 [M+H-C_3_H_3_O_2_]^+^	Coumarin
5	8.29	Cimifugin	C_16_H_18_O_6_	[M+H]^+^	307.1176	307.1172	−1.30	289.1070 [M+H-H_2_O]^+^ 274.0825 [M+H-CH_5_O]^+^ 259.0599 [M+H-C_2_H_8_O]^+^ 247.0597 [M+H-C_3_H_8_O]^+^ 235.0599 [M+H-C_4_H_8_O]^+^ 221.0443 [M+H-C_5_H_10_O]^+^ 205.0495 [M+H-C_5_H_10_O_2_]^+^	Chromone
6	8.33	Fraxidin	C_11_H_10_O_5_	[M+H]^+^	223.0600	223.0597	−1.34	207.0286 [M+H-CH_4_]^+^ 190.0259 [M+H-CH_5_O]^+^ 179.0337 [M+H-C_2_H_4_O]^+^ 162.0311 [M+H-C_2_H_5_O_2_]^+^ 134.0363 [M+H-C_3_H_5_O_3_]^+^	Coumarin
7	8.65	5-O-Methylvisammioside	C_22_H_28_O_10_	[M+H]^+^	453.1755	453.1748	−1.54	291.1224 [M+H-C_6_H_10_O_5_]^+^ 273.1118 [M+H-C_6_H_12_O_6_]^+^	Chromone
8	8.90	Isofraxidin	C_11_H_10_O_5_	[M+H]^+^	223.0600	223.0600	0.00	207.0287 [M+H-CH_4_]^+^ 190.0258 [M+H-CH_5_O]^+^ 179.0337 [M+H-C_2_H_4_O]^+^ 162.0311 [M+H-C_2_H_5_O_2_]^+^ 134.0362 [M+H-C_3_H_5_O_3_]^+^	Coumarin
9	9.93	Khellactone	C_14_H_14_O_5_	[M+H]^+^	263.0914	263.0912	−0.76	203.0702 [M+H-C_2_H_4_O_2_]^+^ 175.0389 [M+H-C_3_H_8_O_2_]^+^	Coumarin
10	10.53	Norcimifugin	C_15_H_16_O_6_	[M+H]^+^	293.1019	293.1017	−0.68	275.0910 [M+H-H_2_O]^+^ 260.0677 [M+H-CH_5_O]^+^ 245.0450 [M+H-C_2_H_8_O]^+^ 233.0444 [M+H-C_3_H_8_O]^+^ 221.0443 [M+H-C_4_H_8_O]^+^	Chromone
11	10.61	5-O-Methylvisamminol	C_16_H_18_O_5_	[M+H]^+^	291.1227	291.1222	−1.72	273.1119 [M+H-H_2_O]^+^ 258.0884 [M+H-CH_5_O]^+^ 243.0650 [M+H-C_2_H_8_O]^+^ 231.0653 [M+H-C_3_H_8_O]^+^ 219.0650 [M+H-C_4_H_8_O]^+^ 205.0494 [M+H-C_5_H_10_O]^+^ 189.0544 [M+H-C_5_H_10_O_2_]^+^	Chromone
12	10.96	Sec-O-Glucosylhamaudol	C_21_H_26_O_10_	[M+H]^+^	439.1598	439.1592	−1.37	277.1068 [M+H-C_6_H_10_O_5_]^+^ 259.0962 [M+H-C_6_H_12_O_6_]^+^ 241.0859 [M+H-C_6_H_14_O_7_]^+^ 217.0493 [M+H-C_9_H_18_O_6_]^+^ 205.0495 [M+H-C_10_H_18_O_6_]^+^	Chromone
13	11.21	Clemiscosin A	C_20_H_18_O_8_	[M+H]^+^	387.1074	387.1069	−1.29	207.0286 [M+H-C_4_H_8_O]^+^ 179.0337 [M+H-C_5_H_8_O_2_]^+^	Coumarin
14	12.41	Psoralen	C_11_H_6_O_3_	[M+H]^+^	187.0389	187.0389	0.00	159.0435 [M+H-CO]^+^ 143.0491 [M+H-CO_2_]^+^ 131.0492 [M+H-C_2_O_2_]^+^ 115.0545 [M+H-C_2_O_3_]^+^	Coumarin
15	12.97	Bergapten	C_12_H_8_O_4_	[M+H]^+^	217.0495	217.0494	−0.46	202.0260 [M+H-CH_3_]^+^ 174.0311 [M+H-C_2_H_3_O]^+^	Coumarin
16	14.04	Hamaudol	C_15_H_16_O_5_	[M+H]^+^	277.1070	277.1067	−1.08	259.0964 [M+H-H_2_O]^+^ 241.0858 [M+H-H_4_O_2_]^+^ 217.0496 [M+H-C_3_H_8_O]^+^ 205.0496 [M+H-C_4_H_8_O]^+^	Chromone
17	14.11	Divaricatol	C_17_H_18_O_7_	[M+H]^+^	335.1125	335.1119	−1.79	275.0912 [M+H-C_2_H_3_O_2_]^+^ 257.0805 [M+H-C_2_H_5_O_3_]^+^ 247.0965 [M+H-C_3_H_3_O_3_]^+^ 233.0444 [M+H-C_5_H_9_O_2_]^+^ 221.0443 [M+H-C_6_H_9_O_2_]^+^ 205.0495 [M+H-C_6_H_9_O_3_]^+^	Chromone
18	17.31	5-methoxy-7-(3,3-dimethylallyloxy)-coumarin	C_15_H_16_O_4_	[M+H]^+^	261.1121	261.1120	−0.38	205.0495 [M+H-C_4_H_8_]^+^ 177.0546 [M+H-C_5_H_8_O]^+^	Coumarin
19	18.02	3′-O-acetylhamaudol	C_17_H_18_O_6_	[M+H]^+^	319.1176	319.1172	−1.25	259.0964 [M+H-C_2_H_3_O_2_]^+^ 231.1014 [M+H-C_3_H_3_O_3_]^+^ 217.0496 [M+H-C_5_H_9_O_2_]^+^ 205.0497 [M+H-C_6_H_9_O_2_]^+^	Chromone
20	18.15	Imperatorin	C_16_H_14_O_4_	[M+H]^+^	271.0964	271.0961	−1.11	203.0339 [M+H-C_5_H_8_]^+^ 175.0390 [M+H-C_6_H_8_O]^+^ 147.0441 [M+H-C_7_H_8_O_2_]^+^	Coumarin
21	18.40	Ledebouriellol	C_20_H_22_O_7_	[M+H]^+^	375.1438	375.1435	−0.80	275.0912 [M+H-C_5_H_8_O_2_]^+^ 233.0444 [M+H-C_7_H_14_O]^+^ 221.0443 [M+H-C_8_H_14_O]^+^	Chromone
22	18.89	Phellopterin	C_17_H_16_O_5_	[M+H]^+^	301.1070	301.1067	−1.00	233.0443 [M+H-C_5_H_8_]^+^ 218.0209 [M+H-C_6_H_11_]^+^	Coumarin
23	19.36	Deltoin	C_19_H_20_O_5_	[M+H]^+^	329.1383	329.1378	−1.52	229.0858 [M+H-C_5_H_8_O_2_]^+^ 214.0621 [M+H-C_6_H_11_O_2_]^+^	Coumarin
24	21.59	Osthenol	C_14_H_14_O_3_	[M+H]^+^	231.1015	231.1012	−1.30	163.0389 [M+H-C_5_H_8_]^+^ 135.0439 [M+H-C_6_H_8_O]^+^ 107.0495 [M+H-C_7_H_8_O_2_]^+^	Coumarin
25	21.97	7-Hydroxycoumarine	C_9_H_6_O_3_	[M+H]^+^	163.0389	163.0385	−2.45	135.0442 [M+H-CO]^+^ 119.0496 [M+H-CO_2_]^+^ 107.0495 [M+H-C_2_O_2_]^+^	Coumarin
26	22.55	3′-O-angeloylhamaudol	C_20_H_22_O_6_	[M+H]^+^	359.1489	359.1481	−2.23	259.0963 [M+H-C_5_H_8_O_2_]^+^ 241.0856 [M+H-C_5_H_10_O_3_]^+^ 231.1013 [M+H-C_6_H_8_O_3_]^+^ 217.0494 [M+H-C_8_H_14_O_2_]^+^ 205.0495 [M+H-C_9_H_14_O]^+^	Chromone

**Table 2:** Identification of metabolites in vivo from *Saposhnikoviae Radix* (positive ion mode).

NO	tR (min)	Formula	Theoretical (*m*/*z*)	Experimental (*m*/*z*)	Δppm	Parent Compound	Biotransformation	Fragment Ion (*m*/*z*)	VIP
M1	6.07	C_16_H_18_O_7_	323.1125	323.1122	−0.93	Cimifugin	Oxidation	305.1019 [M+H-H_2_O]^+^ 259.0604 [M+H-C_2_H_8_O_2_]^+^ 247.0602 [M+H-C_3_H_8_O_2_]^+^ 235.0600 [M+H-C_4_H_8_O_2_]^+^ 221.0442 [M+H-C_5_H_10_O_2_]^+^	1.48
M2	6.45	C_16_H_18_O_7_	323.1125	323.1124	−0.31	Cimifugin	Oxidation	305.0998 [M+H-H_2_O]^+^ 259.0617 [M+H-C_2_H_8_O_2_]^+^ 247.0601 [M+H-C_3_H_8_O_2_]^+^ 235.0599 [M+H-C_4_H_8_O_2_]^+^ 232.0364 [M+H-C_4_H_11_O_2_]^+^ 221.0452 [M+H-C_5_H_10_O_2_]^+^	1.48
M3	6.96	C_16_H_16_O_7_	321.0968	321.0966	−0.62	Divaricatacid	Parent	303.0862 [M+H-H_2_O]^+^ 273.0391 [M+H-C_2_H_8_O]^+^ 249.0392 [M+H-C_3_H_5_O]^+^ 235.0236 [M+H-C_4_H_7_O]^+^	1.45
M4	7.09	C_22_H_28_O_11_	469.1704	469.1702	−0.43	Prim-O-glucosylcimifugin	Parent	307.1175 [M+H-C_6_H_10_O_5_]^+^ 289.1076 [M+H-C_6_H_12_O_6_]^+^	1.42
M5	7.19	C_22_H_26_O_12_	483.1497	483.1493	−0.83	Cimifugin	Glucuronide conjugation	307.1174 [M+H-C_6_H_8_O_6_]^+^ 289.1059 [M+H-C_6_H_10_O_7_]^+^	1.52
M6	7.27	C_16_H_18_O_7_	323.1125	323.1124	−0.31	Cimifugin	Oxidation	305.1014 [M+H-H_2_O]^+^ 275.0543 [M+H-C_2_H_8_O]^+^ 247.0602 [M+H-C_3_H_8_O_2_]^+^ 232.0365 [M+H-C_4_H_11_O_2_]^+^	1.48
M7	8.29	C_16_H_18_O_6_	307.1176	307.1173	−0.98	Cimifugin	Parent	289.1067 [M+H-H_2_O]^+^ 274.0819 [M+H-CH_5_O]^+^ 259.0600 [M+H-C_2_H_8_O]^+^ 247.0599 [M+H-C_3_H_8_O]^+^ 235.0600 [M+H-C_4_H_8_O]^+^ 221.0444 [M+H-C_5_H_10_O]^+^ 205.0494 [M+H-C_5_H_10_O_2_]^+^	1.57
M8	8.36	C_16_H_16_O_6_	305.1017	305.1020	0.98	Cimifugin	Desaturation	289.1065 [M+H-O]^+^ 274.0829 [M+H-CH_3_O]^+^ 259.0598 [M+H-C_2_H_6_O]^+^ 247.0596 [M+H-C_3_H_6_O]^+^ 235.0598 [M+H-C_4_H_6_O]^+^ 221.0442 [M+H-C_5_H_8_O]^+^ 205.0493 [M+H-C_5_H_8_O_2_]^+^	1.55
M9	8.65	C_22_H_28_O_10_	453.1755	453.1754	−0.22	5-O-Methylvisammioside	Parent	291.1225 [M+H-C_6_H_10_O_5_]^+^ 273.1121 [M+H-C_6_H_12_O_6_]^+^	1.48
M10	10.53	C_15_H_16_O_6_	293.1019	293.1017	−0.68	Norcimifugin	Parent	275.0911 [M+H-H_2_O]^+^ 260.0692 [M+H-CH_5_O]^+^ 233.0447 [M+H-C_3_H_8_O]^+^ 221.0444 [M+H-C_4_H_8_O]^+^	1.26
M11	10.61	C_16_H_18_O_5_	291.1227	291.1225	−0.69	5-O-Methylvisamminol	Parent	273.1123 [M+H-H_2_O]^+^ 243.0649 [M+H-C_2_H_8_O]^+^ 219.0650 [M+H-C_4_H_8_O]^+^ 205.0491 [M+H-C_5_H_10_O]^+^	1.34
M12	10.96	C_21_H_26_O_10_	439.1598	439.1595	−0.68	Sec-O-Glucosylhamaudol	Parent	277.1069 [M+H-C_6_H_10_O_5_]^+^ 259.0965 [M+H-C_6_H_12_O_6_]^+^ 217.0494 [M+H-C_9_H_18_O_6_]^+^ 205.0497 [M+H-C_10_H_18_O_6_]^+^	1.42
M13	12.21	C_14_H_14_O_5_	263.0914	263.0912	−0.76	Cimifugin	Loss of CH_2_O + Demethylation	163.039 [M+H-C_5_H_8_O_2_]^+^ 135.0437 [M+H-C_6_H_8_O_3_]^+^ 119.0495 [M+H-C_6_H_8_O_4_]^+^ 107.0497 [M+H-C_7_H_8_O_4_]^+^	1.42
M14	14.04	C_15_H_16_O_5_	277.1070	277.1068	−0.72	Hamaudol	Parent	259.0974 [M+H-H_2_O]^+^ 241.0866 [M+H-H_4_O_2_]^+^ 217.0498 [M+H-C_3_H_8_O]^+^ 205.0496 [M+H-C_4_H_8_O]^+^	1.34

## Data Availability

The original contributions presented in this study are included in the article and Supplementary Materials. Further inquiries can be directed to the corresponding authors.

## Supplementary Materials

The following supporting information can be downloaded at: https://www.mdpi.com/article/10.3390/ijms26157088/s1.

## Abbreviations

The following abbreviations are used in this manuscript:

AIA	Adjuvant induced rheumatoid arthritis
BC	Betweenness centrality
CC	Closeness centrality
DL	Drug-like properties
DMSO	Dimethyl sulfoxide
GO	Gene ontology
KEGG	Kyoto encyclopedia of genes and genomes
LPS	Lipopolysaccharide
MSR	Model mice were given SR
MTT	Methyl thiazolyl tetrazolium
NO	Nitric oxide
NSR	Normal mice were given SR
OB	Oral bioavailability
OPLS-DA	Orthogonal partial least squares discriminant analysis
PCA	Principal component analysis
RA	Rheumatoid arthritis
SR	*Saposhnikoviae Radix*
UPLC-MS/MS	Ultra-performance liquid chromatography/tandem mass spectrometry
VIP	variable importance in projection
