# Supplementary material for: Anti-Inflammatory Pharmacological Mechanism Mediated by the Conversion of Glycosides to Aglycones in Fangfeng (Saposhnikoviae Radix) in Rheumatoid Arthritis Models Based on Serum Metabolomics, Network Pharmacology, and Molecular Docking
Source: Int J Mol Sci. 2025 Jul 23;26(15):7088. doi: 10.3390/ijms26157088 (PMC12346335; doi:10.3390/ijms26157088)
Supplement: Supplementary file 1 [file ijms-26-07088-s001.zip › ijms-3743469-supplementary.pdf]

Supporting information

Anti-Inflammatory Pharmacological Mechanism Mediated by the Conversion of Glycosides to Aglycones in Fangfeng (*Saposhnikoviae Radix*) in Rheumatoid Arthritis Models Based on Serum Metabolomics, Network Pharmacology, and Molecular Docking

Wenguang Jing [1,†], Xiaoyu Lin [2,†], Wenmin Pi [2], Fangliang He [1,2], Haonan Wu [1], Xianrui Wang [1], Jia Chen [1], Xianlong Cheng [1], Penglong Wang [2,*] and Feng Wei [1,*]

[1] *National Institutes for Food and Drug Control, Beijing 102629, China*

[2] *School of Chinese Pharmacy, Beijing University of Chinese Medicine, Beijing 102488, China*

[†] *These authors contributed equally to this work.*

[*] *Corresponding author*

Penglong Wang: wangpenglong@bucm.edu.cn and wpl581@126.com

Feng Wei: weifeng@nifdc.org.cn

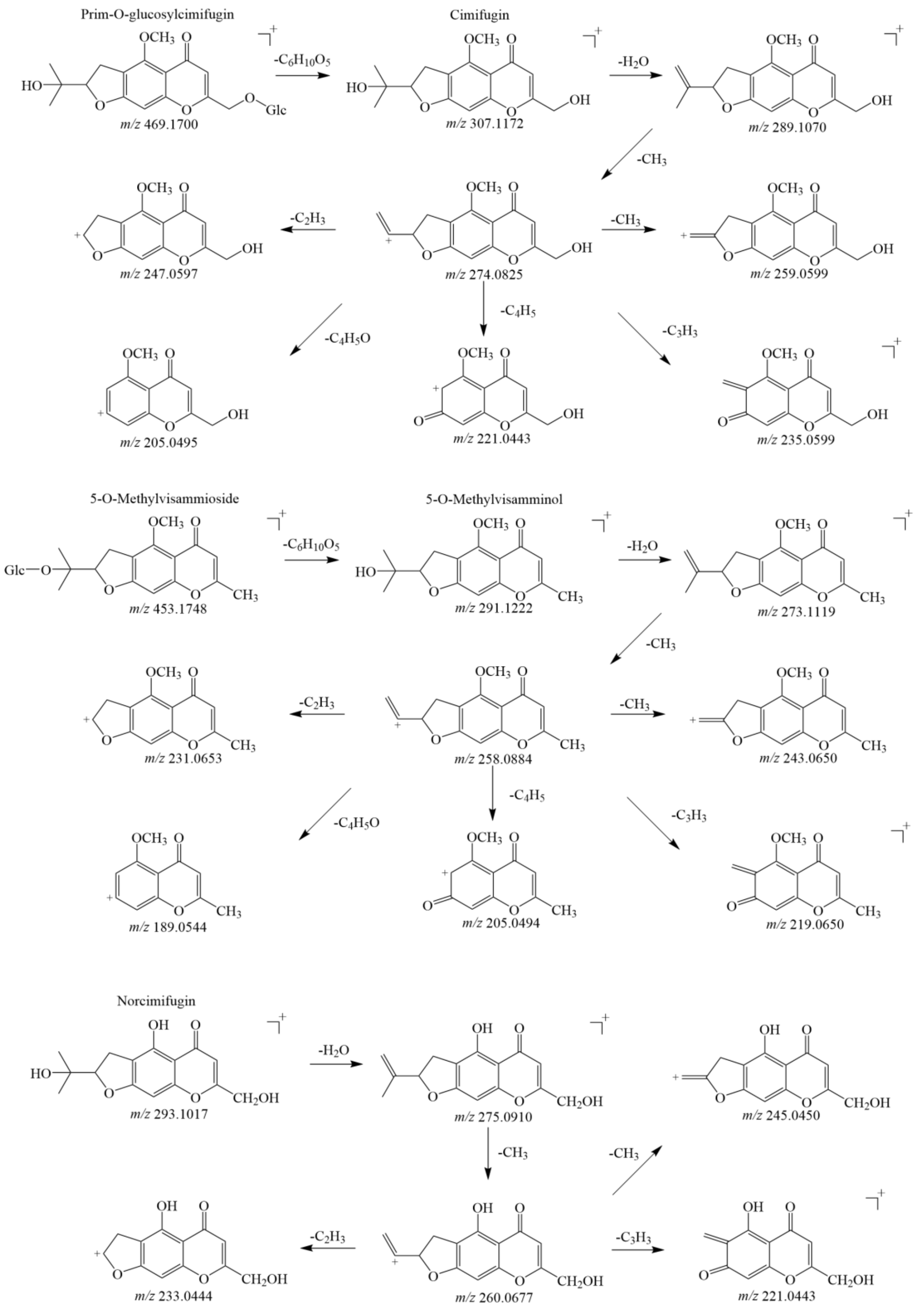

Figure S1. The cracking law of furanochromone.

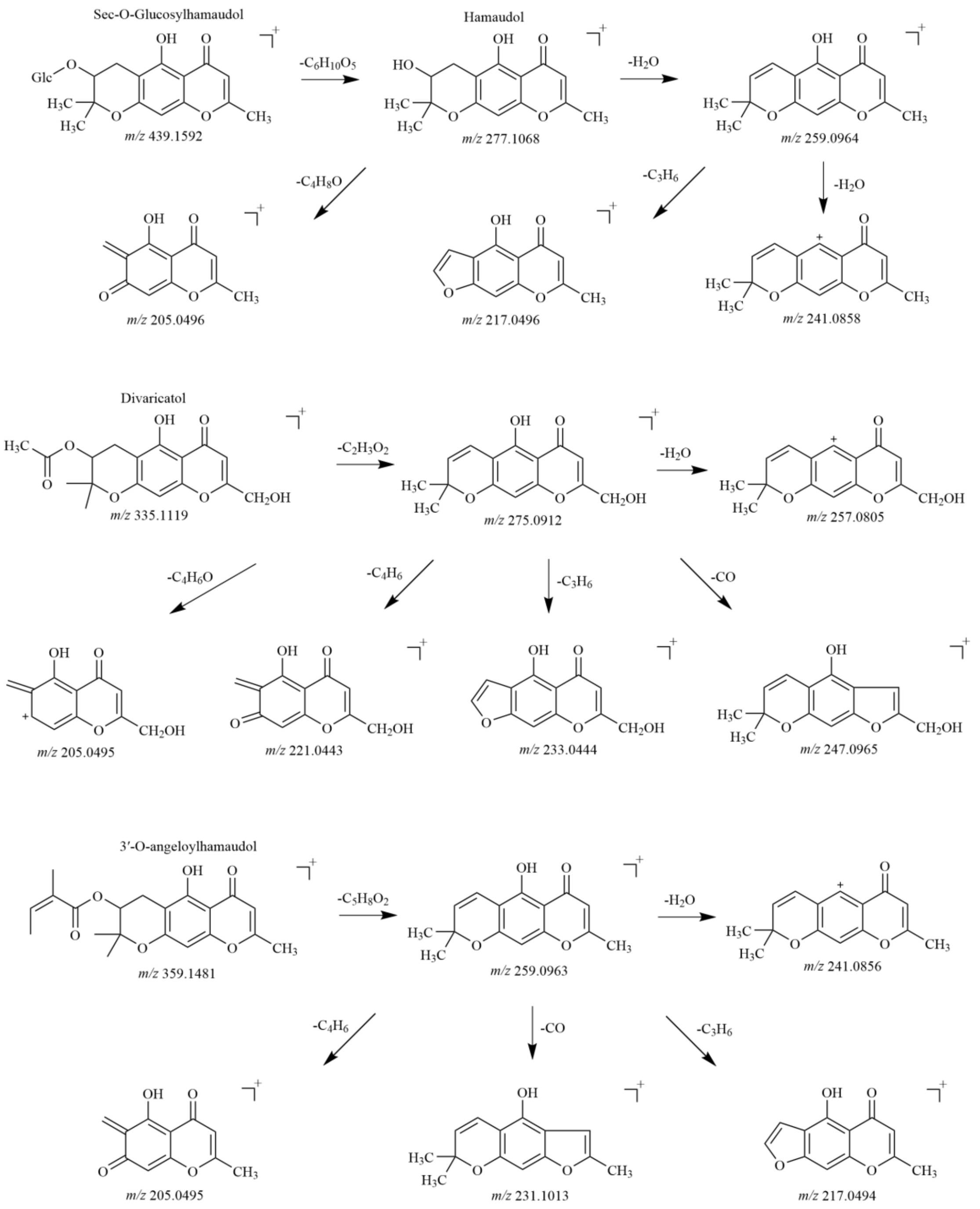

Figure S2. The cracking law of pyranochromone.

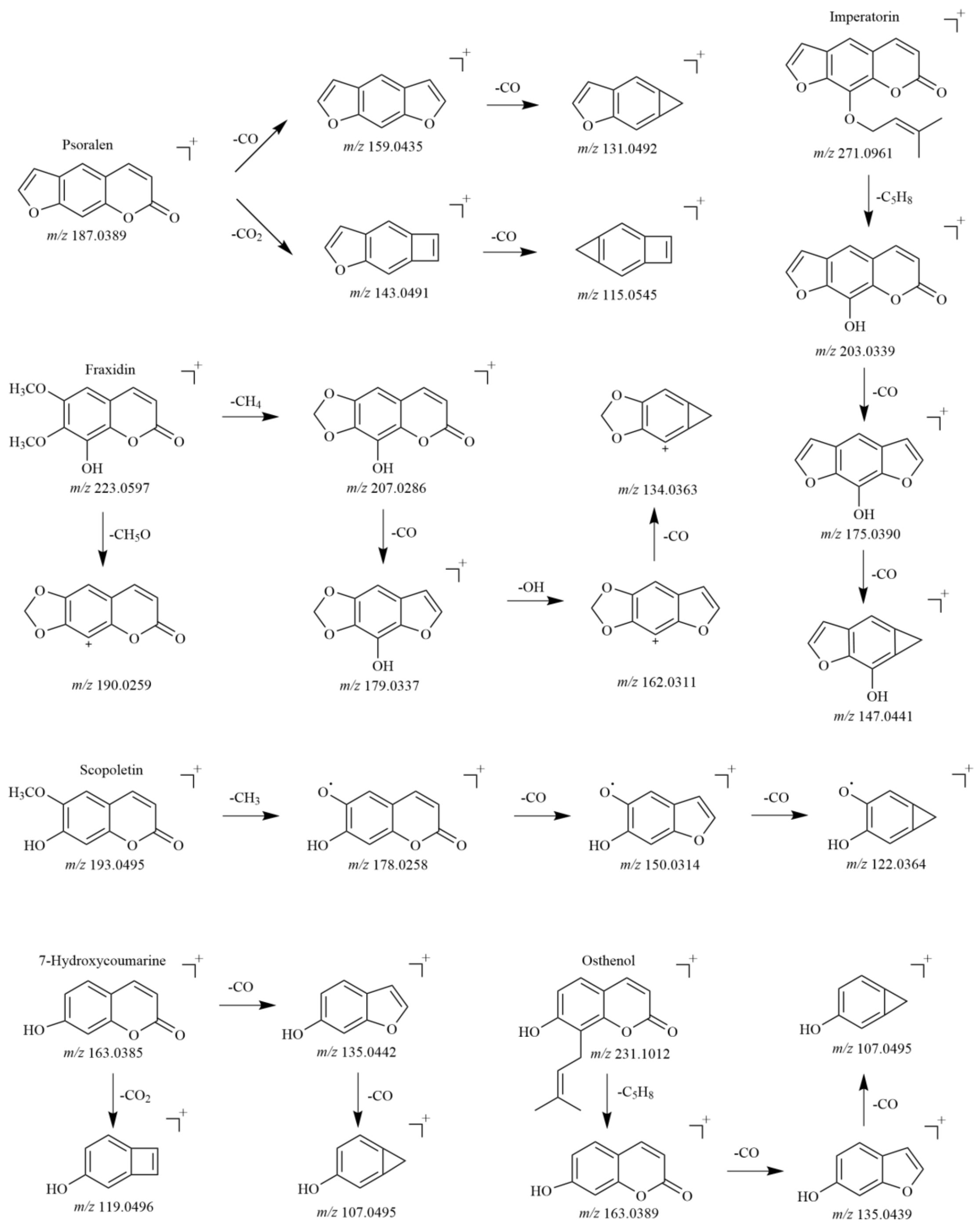

Figure S3. The cracking law of coumarins.

Table S1. Analysis of endogenous metabolites in NSR group and MSR group

Time (min)	Fragment ion	Match	Matched.Form	KEGG	HMDB	PubChem	Normal vs Model			Model vs MSR		
							VIP	FC	Trend	VIP	FC	Trend
0.13	163.1119	Floxuridine	M-HCOOK+H[1+]	C11736	HMDB0014467	5790	1.30	2.70	up	1.20	0.42	down
0.25	149.0446	D-Xylono-1,5-lactone	M+H[1+]	C02266	HMDB0011676	439692	1.08	2.41	up	1.10	0.30	down
0.35	342.9974	D-Fructose 2,6-bisphosphate	M(S34)+H[1+]	C00665	HMDB0001047	105021	1.25	8.29	up	1.11	0.26	down
0.79	160.1807	Aminopropylcadaverine	M+H[1+]	C16565	HMDB0012189	65523	1.06	0.05	down	1.38	156.11	up
0.81	318.9370	Phosphoribosyl pyrophosphate	M-$C_3H_4O_2$+H[1+]	C00119	HMDB0000280	7339	1.43	0.04	down	1.22	15.62	up
0.82	147.1127	Lysine	M+H[1+]	C00047	HMDB0000182	5962	1.46	0.13	down	1.33	7.73	up
0.86	624.8462	myo-Inositol hexakisphosphate	M-H_4O_2+H[1+]	C01204	HMDB0003502	890	1.21	0.41	down	1.08	2.28	up
0.88	673.7774	Liothyronine	M+Na[1+]	C02465	HMDB0000265	5920	1.38	0.20	down	1.26	4.77	up
0.89	279.1289	Didemethylcitalopram	M-H_2O+H[1+]	C16609	HMDB0060472	162976	1.45	0.03	down	1.19	21.33	up
0.92	175.1189	L-Arginine	M+H[1+]	C00062	HMDB0000517	6322	1.11	0.43	down	1.08	2.39	up
0.96	198.0850	Citrulline	M+Na[1+]	C00327	HMDB0000904	9750	1.39	0.25	down	1.33	3.78	up
0.97	137.0710	1-Methylnicotinamide	M[1+]	C02918	HMDB0000699	457	1.25	2.69	up	1.17	0.38	down
0.99	160.0370	Oxoadipic acid	M[1+]	C00322	HMDB0000225	71	1.28	0.37	down	1.21	15.87	up
1.00	114.0665	Creatine	M-H_2O+H[1+]	C00300	HMDB0000064	586	1.07	3.12	up	1.05	0.24	down
1.01	244.0790	N-Acetylgalactosamine	M+Na[1+]	C01132	HMDB0000212	84265	1.44	0.30	down	1.34	3.57	up
1.02	365.1050	Sucrose	M+Na[1+]	C00089	HMDB0000258	5988	1.41	0.19	down	1.26	13.19	up
1.03	130.0863	Pipecolic acid	M+H[1+]	C00408	HMDB0000070	849	1.27	0.34	down	1.31	3.60	up
1.04	146.0923	4-Guanidinobutanoic acid	M+H[1+]	C01035	HMDB0003464	500	1.08	16.36	up	1.22	3.46	up
1.05	268.1037	Adenosine	M+H[1+]	C00212	HMDB0000050	60961	1.00	0.26	down	1.30	28.43	up
1.09	166.9434	2,2,2-Trichloroethanol	M+H_2O+H[1+]	C07490	HMDB0041796	8259	1.32	10.34	up	1.25	0.06	down
1.10	151.0478	D-4'-Phosphopantothenate	M(C13)+2H[2+]	C03492	HMDB0001016	41635	1.39	0.16	down	1.30	9.35	up
1.14	115.0505	L-Asparagine	M-H_2O+H[1+]	C00152	HMDB0000168	6267	1.37	0.35	down	1.24	2.98	up

1.19	205.9879	L-Cysteine	M+HCOOK[1+]	C00097	HMDB0000574	5862	1.18	16.27	up	1.11	0.06	down
1.22	287.0628	Glycineamideribotide	M+H[1+]	C03838	HMDB0002022	160913	1.18	0.00	down	1.13	26.10	up
1.24	110.0717	Histidine	M-HCOOH+H[1+]	C00135	HMDB0000177	6274	1.39	0.26	down	1.30	3.60	up
1.40	109.0763	1-Methylhistamine	M-NH3+H[1+]	C05127	HMDB0000898	3614	1.42	0.21	down	1.28	3.61	up
1.45	166.0532	4-Hydroxy-L-glutamic acid	M(Cl37)+H[1+]	C05947	HMDB0002273	440854	1.38	0.31	down	1.33	3.00	up
1.46	133.0491	D-Ribose	M-H2O+H[1+]	C00121	HMDB0000283	5779	1.42	0.16	down	1.34	6.33	up
1.47	139.0502	Urocanic acid	M+H[1+]	C00785	HMDB0000301	736715	1.36	0.11	down	1.21	6.36	up
1.49	150.0584	Methionine	M+H[1+]	C00073	HMDB0000696	6137	1.31	0.23	down	1.15	3.50	up
1.51	168.0655	Pyridoxal	M+H[1+]	C00250	HMDB0001545	1050	1.27	0.08	down	1.34	53.02	up
1.52	123.0555	Niacinamide	M+H[1+]	C00153	HMDB0001406	936	1.19	0.26	down	1.04	3.36	up
1.65	290.1344	Ophthalmic acid	M+H[1+]	C21016	HMDB0005765	7018721	1.39	0.01	down	1.20	130.53	up
1.70	120.0658	L-Threonine	M+H[1+]	C00188	HMDB0000167	6288	1.43	0.01	down	1.28	57.14	up
1.97	238.0933	Sepiapterin	M+H[1+]	C00835	HMDB0000238	65253	1.41	0.14	down	1.37	5.76	up
2.03	147.0760	Glutamine	M+H[1+]	C00064	HMDB0000641	5961	1.33	0.36	down	1.33	2.97	up
2.06	113.0349	Uracil	M+H[1+]	C00106	HMDB0000300	1174	1.46	0.06	down	1.35	13.97	up
2.11	317.1157	7,8-Dihydropteroic acid	M(S34)+H[1+]	C00921	HMDB0001412	170	1.38	0.11	down	1.18	3.89	up
2.13	128.0707	2,3,4,5-Tetrahydro-2-pyridinecarboxylic acid	M+H[1+]	C00450	HMDB0012130	24771808	1.35	0.49	down	1.29	2.35	up
2.15	233.0599	gamma-Glutamylcysteine	M-H_2O+H[1+]	C00669	HMDB0001049	123938	1.46	0.00	down	1.25	368.69	up
2.16	271.0289	2-Oxo-4-hydroxy-4-carboxy-5-ureidoimidazoline	M+HCOONa[1+]	C12248	HMDB0059663	101957722	1.43	0.01	down	1.29	28.38	up
2.17	182.0812	L-Tyrosine	M+H[1+]	C00082	HMDB0000158	6057	1.45	0.16	down	1.36	4.79	up
2.25	447.2022	Estrone glucuronide	M+H[1+]	C11133	HMDB0004483	115255	1.40	0.13	down	1.13	10.06	up
2.39	163.1229	N-Methylserotonin	M-CO+H[1+]	C06212	HMDB0004369	150885	1.37	0.34	down	1.21	2.60	up
2.43	116.0529	Melibiitol	M(C13)+3H[3+]	C05399	HMDB0006791	440655	1.29	0.25	down	1.31	2.82	up
2.48	251.0637	Deoxyuridine	M+Na[1+]	C00526	HMDB0000012	13712	1.44	0.17	down	1.30	7.86	up

2.52	154.0499	3-Hydroxyanthranilic acid	M+H[1+]	C00632	HMDB0001476	86	1.41	0.06	down	1.30	8.09	up
2.53	175.0478	N-Acetyl-L-aspartic acid	M[1+]	C01042	HMDB0000812	65065	1.38	0.00	down	1.31	89.05	up
2.62	153.0658	Nudifloramide	M+H[1+]	C05842	HMDB0004193	69698	1.40	0.05	down	1.30	9.60	up
2.72	184.0604	4-Pyridoxic acid	M+H[1+]	C00847	HMDB0000017	6723	1.43	0.03	down	1.37	50.75	up
2.90	183.1128	Porphobilinogen	M-CO_2+H[1+]	C00931	HMDB0000245	1021	1.44	0.15	down	1.02	40.51	up
2.92	167.9825	Phosphoenolpyruvic acid	M[1+]	C00074	HMDB0000263	1005	1.39	0.00	down	1.36	352.41	up
3.25	172.0969	Dopamine	M+H_2O+H[1+]	C03758	HMDB0000073	681	1.31	0.03	down	1.30	8.31	up
3.27	190.1073	N(omega)-Hydroxyarginine	M[1+]	C05933	HMDB0004224	43088	1.28	0.04	down	1.23	5.37	up
3.28	209.0920	Kynurenine	M+H[1+]	C00328	HMDB0000684	161166	1.13	0.25	down	1.34	7.97	up
3.32	385.0839	4'-Phosphopantothenoylcysteine	M-H_2O+H[1+]	C04352	HMDB0001117	440304	1.43	0.00	down	1.38	251.86	up
3.33	137.0457	Hypoxanthine	M+H[1+]	C00262	HMDB0000157	790	1.44	0.04	down	1.35	21.32	up
3.38	127.0503	Thymine	M+H[1+]	C00178	HMDB0000262	1135	1.46	0.04	down	1.34	23.52	up
3.41	231.1339	Saccharopine	M-HCOOH+H[1+]	C00449	HMDB0000279	160556	1.27	0.01	down	1.37	60.68	up
3.45	199.1076	Thymidine	M-CO_2+H[1+]	C00214	HMDB0000273	5789	1.44	0.17	down	1.28	7.66	up
3.47	139.0865	N2-Acetylornithine	M-H_4O_2+H[1+]	C00437	HMDB0003357	439232	1.40	0.04	down	1.22	29.59	up
3.59	155.0704	4-Hydroxyphenylacetaldehyde	M+H_2O+H[1+]	C03765	HMDB0003767	440113	1.46	3.19	up	1.34	0.46	down
3.73	220.1179	Pantothenic acid	M+H[1+]	C00864	HMDB0000210	6613	1.08	0.15	down	1.36	10.88	up
4.11	146.1176	4-Trimethylammoniobutanoic acid	M[1+]	C01181	HMDB0001161	725	1.40	0.15	down	1.25	81.38	up
4.55	136.0618	Adenine	M+H[1+]	C00147	HMDB0000034	190	1.44	0.03	down	1.38	39.56	up
4.69	237.0867	L-Formylkynurenine	M+H[1+]	C02700	HMDB0060485	25202092	1.36	0.06	down	1.31	25.34	up
4.70	117.0575	3,4-Dihydroxymandelic acid	M-HCOONa+H[1+]	C05580	HMDB0001866	85782	1.40	0.06	down	1.35	15.03	up
4.72	192.0656	5-Hydroxyindoleacetic acid	M+H[1+]	C05635	HMDB0000763	1826	1.21	0.29	down	1.18	3.91	up
4.78	195.1132	Serotonin	M+H_2O+H[1+]	C00780	HMDB0000259	5202	1.31	0.15	down	1.30	3.86	up
5.12	124.0395	Nicotinic acid	M+H[1+]	C00253	HMDB0001488	938	1.39	2.36	up	1.33	0.40	down
5.41	135.0440	p-Hydroxyphenylacetic acid	M-H_2O+H[1+]	C00642	HMDB0000020	127	1.34	0.15	down	1.03	2.36	up
5.44	251.0484	Alcophosphamide	M-CO+H[1+]	C16551	HMDB0060432	98612	1.04	0.03	down	1.10	15.54	up

5.59	193.0495	3-Carbamoyl-2-phenylpropionic acid	M-NH_3+H[1+]	C16591	HMDB0060367	3025746	1.46	9.49	up	1.19	8.50	up
5.86	214.1073	2-Phenyl-1,3-propanediol monocarbamate	M+H_2O+H[1+]	C16586	HMDB0060351	213060	1.15	0.02	down	1.27	12.87	up
5.88	148.0392	Indole-5,6-quinone	M+H[1+]	C05579	HMDB0006779	440728	1.13	0.27	down	1.32	9.65	up
5.94	174.0550	1-nitronaphthalene	M+H[1+]	C14040	HMDB0062188	6849	1.36	0.08	down	1.32	20.79	up
6.09	274.1106	trans-3,4-Dihydro-3,4-dihydroxy-7,12-dimethylbenz[a]anthracene	M-NH_3+H[1+]	C19490	HMDB0060517	156317	1.34	0.11	down	1.30	8.26	up
6.11	331.1650	Coproporphyrinogen III	M+2H[2+]	C03263	HMDB0001261	321	1.43	0.00	down	1.34	51386.00	up
6.53	227.0847	L-3-Hydroxykynurenine	M(Cl37)+H[1+]	C03227	HMDB0011631	11811	1.10	0.01	down	1.08	236.22	up
6.69	194.0813	Phenylacetylglycine	M+H[1+]	C05598	HMDB0000821	68144	1.37	0.09	down	1.11	10.87	up
7.70	212.1646	Calcidiol	M+H+Na[2+]	C01561	HMDB0003550	5283731	1.30	0.01	down	1.27	92.53	up
7.83	120.0810	Phenylalanine	M-HCOOH+H[1+]	C00079	HMDB0000159	6140	1.43	0.38	down	1.28	3.32	up
8.45	140.0681	L-Valine	M+Na[1+]	C00183	HMDB0000883	6287	1.43	0.47	down	1.27	2.27	up
8.46	201.1024	N-Acetylserotonin	M-H_2O+H[1+]	C00978	HMDB0001238	903	1.47	0.14	down	1.20	8.84	up
8.77	277.1281	Glycerophosphocholine	M+H_2O+H[1+]	C00670	HMDB0000086	657272	1.42	0.00	down	1.36	5641.50	up
8.94	251.1025	Deoxyadenosine	M[1+]	C00559	HMDB0000101	13730	1.40	0.00	down	1.34	1762.40	up
8.97	131.0492	Homovanillin	M-H_4O_2+H[1+]	C05581	HMDB0005175	151276	1.30	0.00	down	1.14	215.94	up
9.51	220.2174	gamma-Glutamylalanine	M(C13)+H[1+]	C03740	HMDB0006248	440103	1.43	0.07	down	1.34	10.05	up
9.70	187.0867	L-Tryptophan	M-H_2O+H[1+]	C00078	HMDB0000929	6305	1.47	0.00	down	1.38	31007.00	up
9.84	601.2652	Biliverdin	M+H_2O+H[1+]	C00500	HMDB0001008	5353439	1.26	0.11	down	1.19	11.51	up
10.48	301.1182	Pantetheine	M+Na[1+]	C00831	HMDB0003426	479	1.45	0.00	down	1.32	36218.00	up
11.53	464.2829	Taurochenodesoxycholic acid	M-H_4O_2+H[1+]	C05465	HMDB0000951	387316	1.11	0.04	down	1.04	23.48	up
12.17	363.2162	Cortisol	M+H[1+]	C00735	HMDB0000063	5754	1.15	0.02	down	1.35	78.68	up

12.28	291.0863	Melatonin	M+NaCl[1+]	C01598	HMDB0001389	896	1.13	64.05	up	1.11	52.09	up
12.36	387.2145	Dihydrocortisol	M+Na[1+]	C05471	HMDB0003259	164838	1.21	3181.20	up	1.13	0.22	down
12.66	315.1336	Chitin	M(C13)+2H[2+]	C00461	HMDB0003362	444514	1.42	0.14	down	1.34	6.60	up
12.72	345.2059	11-Dehydrocorticosterone	M+H[1+]	C05490	HMDB0004029	13783449	1.23	0.19	down	1.36	7.01	up
13.21	347.2216	Corticosterone	M+H[1+]	C02140	HMDB0001547	5753	1.15	0.41	down	1.21	2.73	up
13.26	120.0445	2-Aminobenzoic acid	M-H_2O+H[1+]	C00108	HMDB0001123	227	1.45	0.03	down	1.28	72.16	up
13.28	162.0549	Quinoline-4,8-diol	M+H[1+]	C05637	HMDB0060289	440737	1.30	0.00	down	1.30	732.82	up
13.48	267.1225	Thiamine	M(C13)+H[1+]	C00378	HMDB0000235	1130	1.15	0.22	down	1.18	12.14	up
14.79	156.1206	All-trans-13,14-dihydroretinol	M+H+Na[2+]	C15492	HMDB0011618	446798	1.14	8.87	up	1.01	0.22	down
15.17	318.3001	Phytosphingosine	M+H[1+]	C12144	HMDB0004610	122121	1.43	0.22	down	1.30	3.61	up
15.42	113.0600	Ketoleucine	M-H_2O+H[1+]	C00233	HMDB0000695	70	1.41	0.30	down	1.32	3.10	up
15.85	433.3306	3beta,7alpha-Dihydroxy-5-cholestenoate	M+H[1+]	C17335	HMDB0012454	3081084	1.01	0.10	down	1.24	136.79	up
15.92	275.1253	1a,11b-Dihydro-4,9-dimethylbenz[a]anthra[3,4-b]oxirene	M(Cl37)+H[1+]	C19489	HMDB0060341	53297434	1.21	0.12	down	1.14	9.38	up
16.03	260.2377	Thyroxine	M(C13)+3H[3+]	C01829	HMDB0000248	5819	1.11	15.28	up	1.03	0.08	down
16.21	150.0301	7-Methylxanthine	M-NH_3+H[1+]	C16353	HMDB0001991	68374	1.35	52.93	up	1.22	0.16	down
16.51	616.1762	Heme	M[1+]	C00032	HMDB0003178	26945	1.21	0.00	down	1.25	32456.00	up
16.87	185.0238	1,2-Dihydroxy-3-keto-5-methylthiopentene	M+Na[1+]	C15606	HMDB0012134	5462190	1.29	3.51	up	1.28	0.08	down
16.89	354.2401	11,12,15-THETA	M[1+]	C14782	HMDB0004684	11954043	1.46	0.01	down	1.13	20.47	up
17.01	129.0546	L-Fucose	M-H_4O_2+H[1+]	C01019	HMDB0000174	17106	1.40	0.11	down	1.36	7.33	up
17.10	438.3209	Glycocholic acid	M-CO+H[1+]	C01921	HMDB0000138	10140	1.42	0.01	down	1.22	65.24	up
17.26	302.3052	Sphinganine	M+H[1+]	C00836	HMDB0000269	91486	1.43	0.34	down	1.31	2.46	up

17.58	209.1171	4-(Methylnitrosamino)-1-(3-pyridyl)-1-butanol	M[1+]	C19574	HMDB0041809	104856	1.18	0.17	down	1.35	6.16	up
17.79	300.2896	Sphingosine	M+H[1+]	C00319	HMDB0000252	5280335	1.31	0.41	down	1.18	2.26	up
17.88	380.2553	Sphingosine 1-phosphate	M+H[1+]	C06124	HMDB0000277	5283560	1.34	0.21	down	1.29	9.71	up
17.97	127.0390	Gentisic acid	M-CO+H[1+]	C00628	HMDB0000152	3469	1.32	0.39	down	1.17	2.25	up
18.05	516.3014	Taurocholic acid	M+H[1+]	C05122	HMDB0000036	6675	1.44	0.07	down	1.34	14.27	up
18.27	416.3367	Galactosylsphingosine	M-HCOOH+H[1+]	C01747	HMDB0000648	5280458	1.44	0.09	down	1.05	3.26	up
18.34	182.0602	Pyridoxine 5'-phosphate	M-HCOONa+H[1+]	C00627	HMDB0001319	1055	1.27	22.21	up	1.19	0.04	down
18.35	311.2572	Androstan-3alpha,17beta-diol	M+H_2O+H[1+]	C03852	HMDB0060437	107744	1.44	0.03	down	1.35	10.96	up
18.40	382.2715	Sphinganine 1-phosphate	M+H[1+]	C01120	HMDB0001383	644260	1.30	0.16	down	1.33	17.22	up
18.63	247.2055	Leukotriene A4	M-$C_3H_4O_2$+H[1+]	C00909	HMDB0001337	5280383	1.46	0.01	down	1.18	6.87	up
18.66	476.2407	Retinoyl b-glucuronide	M[1+]	C11061	HMDB0003141	5281877	1.40	0.04	down	1.34	19.81	up
18.89	422.3259	Chenodeoxycholic acid glycine conjugate	M-CO+H[1+]	C05466	HMDB0000637	12544	1.46	0.04	down	1.31	10.85	up
19.13	275.2004	16a-Hydroxyandrost-4-ene-3,17-dione	M-CO+H[1+]	C05140	HMDB0006774	440574	1.47	0.03	down	1.11	3.63	up
19.34	140.0105	Phosphoserine	M-HCOOH+H[1+]	C01005	HMDB0000272	68841	1.47	0.01	down	1.37	71.86	up
19.40	409.0311	2'-Deoxyinosine triphosphate	M-HCOOK+H[1+]	C01345	HMDB0003537	146302	1.21	39.74	up	1.11	0.08	down
19.52	632.2422	Tetrahydrofolyl-[Glu](n)	M-$C_3H_4O_2$+H[1+]	C03541	HMDB0006826	45479706	1.45	0.14	down	1.27	3.78	up
19.58	277.2159	Stearidonic acid	M+H[1+]	C16300	HMDB0006547	5312508	1.47	0.10	down	1.06	2.63	up
19.62	634.1358	4,5-Dihydro-4-hydroxy-5-S-glutathionyl-benzo[a]pyrene	M+NaCl[1+]	C14855	HMDB0060391	11954068	1.36	0.06	down	1.34	12.80	up
20.04	129.0696	Naphthalene	M+H[1+]	C00829	HMDB0029751	931	1.08	5.51	up	1.02	0.18	down
20.19	315.1949	Cortisone	M-HCOOH+H[1+]	C00762	HMDB0002802	222786	1.45	0.01	down	1.27	5.66	up
20.22	159.0443	Indoleacetic acid	M-NH_3+H[1+]	C00954	HMDB0000197	802	1.17	31.70	up	1.08	0.08	down
20.26	446.1616	Dihydrofolic acid	M(Cl37)+H[1+]	C00415	HMDB0001056	98792	1.28	0.07	down	1.35	6.11	up
20.33	153.1386	N6,N6,N6-Trimethyl-L-lysine	M-H_4O_2+H[1+]	C03793	HMDB0001325	440120	1.45	0.17	down	1.37	5.64	up

20.47	169.1221	Leukotriene B4	M+2H[2+]	C02165	HMDB0001085	5280492	1.45	0.18	down	1.33	6.33	up
20.48	127.1117	Caprylic acid	M-H_2O+H[1+]	C06423	HMDB0000482	379	1.43	0.01	down	1.36	106.85	up
20.75	141.1271	Linoleic acid	M+2H[2+]	C01595	HMDB0000673	5280450	1.46	0.18	down	1.35	6.22	up
20.76	356.3523	Palmitoylcarnitine	M-CO_2+H[1+]	C02990	HMDB0000222	11953816	1.21	0.35	down	1.20	2.05	up
20.77	339.2889	5alpha-Pregnane-3alpha,20alpha-diol	M+H_2O+H[1+]	C18042	HMDB0060409	164674	1.43	0.18	down	1.34	3.59	up
20.86	309.2425	Androsterone	M+H_2O+H[1+]	C00523	HMDB0000031	5879	1.16	6.23	up	1.14	0.04	down
20.88	153.1273	Arachidonic acid	M+2H[2+]	C00219	HMDB0001043	444899	1.46	0.15	down	1.37	7.94	up
20.89	279.2316	gamma-Linolenic acid	M+H[1+]	C06426	HMDB0003073	5280933	1.47	0.04	down	1.36	28.18	up
21.57	163.0388	D-Ribose 5-phosphate	M-HCOONa+H[1+]	C00117	HMDB0001548	440101	1.40	5.32	up	1.20	4.20	up
21.70	399.1989	3beta-Hydroxypregn-5-en-20-one sulfate	M(S34)+H[1+]	C18044	HMDB0060382	105074	1.34	0.19	down	1.17	3.30	up
21.92	341.0934	Pantetheine 4'-phosphate	M-H_2O+H[1+]	C01134	HMDB0001416	115254	1.48	0.07	down	1.13	3.80	up
21.93	233.1904	16a-Hydroxydehydroisoandrosterone	M-$C_3H_4O_2$+H[1+]	C05139	HMDB0000352	102030	1.36	2.48	up	1.33	0.24	down
21.99	303.2318	Eicosapentaenoic acid	M+H[1+]	C06428	HMDB0001999	446284	1.18	6.97	up	1.09	0.17	down
22.02	435.1211	(1R)-Hydroxy-(2R)-glutathionyl-1,2-d ihydronaphthalene	M-NH_3+H[1+]	C14791	HMDB0060300	11954044	1.26	12.02	up	1.19	0.06	down
22.64	273.1844	Estradiol	M+H[1+]	C00951	HMDB0000151	5757	1.39	0.04	down	1.11	3.17	up
22.72	149.0233	Formylanthranilic acid	M-NH_3+H[1+]	C05653	HMDB0004089	101399	1.33	2.60	up	1.30	0.21	down
22.76	177.0399	D-Glucurono-6,3-lactone	M+H[1+]	C02670	HMDB0006355	2724333	1.39	3.28	up	1.32	0.24	down
22.77	169.0349	Uric acid	M+H[1+]	C00366	HMDB0000289	1175	1.37	2.46	up	1.30	0.33	down
22.80	151.0754	3-Methoxytyramine	M-NH_3+H[1+]	C05587	HMDB0000022	1669	1.47	2.33	up	1.38	0.37	down
22.81	137.0598	Phenylpyruvic acid	M-CO+H[1+]	C00166	HMDB0000205	997	1.47	2.11	up	1.38	0.43	down
22.83	283.0484	5-Fluorodeoxyuridine monophosphate	M-CO_2+H[1+]	C04242	HMDB0060394	8642	1.32	3.30	up	1.29	0.19	down
22.87	223.0963	4-[(Hydroxymethyl)nitrosoamino]-1-(3-pyridinyl)-1-butanone	M[1+]	C19563	HMDB0062382	53297437	1.39	31.43	up	1.28	0.14	down
23.15	448.1191	Octanoyl-CoA	M(C13)+2H[2+]	C01944	HMDB0001070	380	1.10	4.62	up	1.11	0.07	down

23.39	321.2430	15-HETE	M+H[1+]	C04742	HMDB0003876	5280724	1.07	49.19	up	1.01	0.00	down
24.09	286.1045	Hydroxymethylbilane	M(C13)+3H[3+]	C01024	HMDB0001137	788	1.40	0.23	down	1.32	7.11	up
24.30	223.2059	Farnesol	M+H[1+]	C06081	HMDB0004305	445070	1.25	22.05	up	1.17	0.05	down
24.41	174.9571	4-Bromophenol	M(Cl37)+H[1+]	C14453	HMDB0062397	7808	1.05	2.03	up	1.11	0.37	down
24.57	247.0937	Biotin	M(Cl37)+H[1+]	C00120	HMDB0000030	171548	1.46	10.77	up	1.37	0.18	down
24.73	139.0024	cis-Aconitic acid	M-H_4O_2+H[1+]	C00417	HMDB0000072	643757	1.43	0.12	down	1.37	7.85	up
24.74	129.0182	Oxoglutaric acid	M-H_2O+H[1+]	C00026	HMDB0000208	51	1.44	0.11	down	1.38	9.33	up
24.81	421.2236	Estriol-16-Glucuronide	M-CO_2+H[1+]	C05504	HMDB0006766	122281	1.31	0.02	down	1.36	49.19	up
25.07	127.0755	3,4-Dihydroxyphenylglycol	M-CO_2+H[1+]	C05576	HMDB0000318	91528	1.37	0.01	down	1.05	73.02	up
25.21	443.2057	2-Methoxy-estradiol-17b 3-glucuronide	M-H_4O_2+H[1+]	C11131	HMDB0006765	443076	1.31	3.07	up	1.17	0.35	down
25.39	132.1019	Leucine	M+H[1+]	C00123	HMDB0000687	6106	1.36	0.22	down	1.32	3.99	up
25.48	465.2485	Testosterone glucuronide	M+H[1+]	C11134	HMDB0003193	108192	1.40	0.13	down	1.36	5.67	up
25.51	403.1389	S-Adenosylhomocysteine	M+H_2O+H[1+]	C00021	HMDB0000939	439155	1.20	0.05	down	1.01	3.04	up
25.72	415.3197	Coprocholic acid	M-H_4O_2+H[1+]	C04722	HMDB0000601	122312	1.16	0.09	down	1.36	5.49	up
25.82	151.0241	2-Oxo-4-methylthiobutanoic acid	M(Cl37)+H[1+]	C01180	HMDB0001553	473	1.29	3.17	up	1.22	0.30	down
25.84	188.0922	Norepinephrine	M+H_2O+H[1+]	C00547	HMDB0000216	439260	1.32	4.01	up	1.27	0.21	down
25.95	297.2426	9,10-Epoxyoctadecenoic acid	M+H[1+]	C14825	HMDB0004701	6246154	1.03	10.59	up	1.04	0.00	down
26.24	190.0501	1-Nitronaphthalene-5,6-oxide	M+H[1+]	C14800	HMDB0060331	11954051	1.17	2.03	up	1.25	0.26	down
26.44	329.2469	Docosahexaenoic acid	M+H[1+]	C06429	HMDB0002183	445580	1.32	0.10	down	1.30	18.44	up
26.46	417.3359	Calcitriol	M+H[1+]	C01673	HMDB0001903	5280453	1.28	0.00	down	1.28	371.21	up
26.48	457.3287	3a,7a,12a-Trihydroxy-5b-cholestan-26 -al	M+Na[1+]	C01301	HMDB0003533	439479	1.11	0.04	down	1.22	9.88	up
26.54	161.0601	Naphthalene-1,2-diol	M+H[1+]	C03012	HMDB0060497	11318	1.07	3.19	up	1.12	0.15	down
26.59	190.0716	N-Acetyl-L-glutamic acid	M+H[1+]	C00624	HMDB0001138	70914	1.02	4.06	up	1.07	0.08	down
26.68	156.0423	Betaine	M+K[1+]	C00719	HMDB0000043	247	1.04	3.07	up	1.12	0.15	down

27.04	153.0772	Theobromine	M-CO+H[1+]	C07480	HMDB0002825	5429	1.05	3.83	up	1.12	0.05	down
27.07	545.3096	Urobilinogen	M-HCOOH+H[1+]	C05791	HMDB0004158	440784	1.32	0.14	down	1.10	2.99	up
27.50	291.0782	9-Hydroxybenzo[a]pyrene	M+Na[1+]	C14556	HMDB0062438	28598	1.42	45.35	up	1.32	0.13	down
27.55	289.2163	Testosterone	M+H[1+]	C00535	HMDB0000234	6013	1.06	2.87	up	1.18	0.10	down
27.67	303.2896	Stearic acid	M+H_2O+H[1+]	C01530	HMDB0000827	5281	1.31	5.09	up	1.29	0.07	down
27.75	154.1227	Metanephrine	M-CO_2+H[1+]	C05588	HMDB0004063	21100	1.41	0.07	down	1.35	9.51	up
27.92	144.0809	Tryptamine	M-NH_3+H[1+]	C00398	HMDB0000303	1150	1.31	3.81	up	1.29	0.16	down
28.00	215.1541	3-Hydroxylidocaine	M-H_4O_2+H[1+]	C16560	HMDB0060655	161824	1.46	6.02	up	1.37	0.17	down
28.19	341.0519	Nicotinic acid ribonucleoside	M+HCOOK[1+]	C05841	HMDB0006809	161234	1.18	3.93	up	1.22	0.04	down
28.27	559.1310	CMP-N-glycoloylneuraminate	M-$C_3H_4O_2$+H[1+]	C03691	HMDB0012206	53481387	1.12	2.12	up	1.34	0.15	down
28.43	325.0229	5-Fluorouridine monophosphate	M-H_2O+H[1+]	C16634	HMDB0060397	150856	1.27	8.95	up	1.20	0.11	down
28.58	203.1798	2-trans,6-trans-Farnesal	M-H_2O+H[1+]	C03461	HMDB0060356	5280598	1.14	183.84	up	1.04	0.07	down
29.05	204.0876	Dihydrobiopterin	M-H_4O_2+H[1+]	C00268	HMDB0000038	119055	1.04	2.10	up	1.37	0.03	down
29.87	471.1052	Citicoline	M-H_2O+H[1+]	C00307	HMDB0001413	13805	1.41	4.27	up	1.35	0.14	down
30.08	150.0445	alpha-Fluoro-beta-ureidopropionic acid	M[1+]	C16631	HMDB0060435	151244	1.14	7.01	up	1.07	0.14	down
30.41	275.0491	Acrylyl-CoA	M(C13)+3H[3+]	C00894	HMDB0002307	9543026	1.25	5.96	up	1.23	0.07	down
30.57	206.1033	Tetrahydrobiopterin	M-H_4O_2+H[1+]	C00272	HMDB0000027	44257	1.21	207.55	up	1.12	0.05	down
30.58	1144.3447	Tetracosahexaenoyl CoA	M+K[1+]	C16168	HMDB0006243	53477806	1.15	22.06	up	1.08	0.05	down
30.81	210.0871	Deoxycytidine	M-H_2O+H[1+]	C00881	HMDB0000014	13711	1.43	0.01	down	1.38	51.46	up
30.85	125.1074	Pyridoxamine	M-CO_2+H[1+]	C00534	HMDB0001431	1052	1.47	0.18	down	1.29	6.95	up
30.91	126.0913	Pyridoxine	M-CO_2+H[1+]	C00314	HMDB0000239	1054	1.47	0.16	down	1.38	9.43	up
31.31	268.0317	5-Amino-1-(5-phospho-D-ribosyl)imidazole-4-carboxylic acid	M-$C_3H_4O_2$+H[1+]	C04751	HMDB0006273	165388	1.35	4.70	up	1.30	0.05	down

31.66	369.3512	Cholesterol	M-H_2O+H[1+]	C00187	HMDB0000067	5997	1.03	0.43	down	1.21	2.90	up
31.68	383.3305	7-Dehydrodesmosterol	M+H[1+]	C05107	HMDB0003896	440558	1.29	0.03	down	1.28	50.12	up
31.70	259.1688	2-Hydroxyestrone	M-CO+H[1+]	C05298	HMDB0000343	440623	1.11	0.22	down	1.17	2.86	up
32.00	381.2987	Cholic acid	M-CO+H[1+]	C00695	HMDB0000619	221493	1.46	0.18	down	1.33	5.93	up
32.34	411.3623	4,4-Dimethylcholesta-8,14,24-trienol	M+H[1+]	C11455	HMDB0001023	443212	1.19	133.8<br>8	up	1.12	0.00	down
32.38	243.1744	Estrone	M-CO+H[1+]	C00468	HMDB0000145	5870	1.11	84.75	up	1.04	0.01	down
32.43	327.2524	Prostaglandin F2a	M-CO+H[1+]	C00639	HMDB0001139	5283078	1.33	0.29	down	1.28	2.54	up
32.92	138.0911	Tyramine	M+H[1+]	C00483	HMDB0000306	5610	1.43	0.08	down	1.29	12.35	up
33.18	995.2930	Bilirubin diglucuronide	M+NaCl[1+]	C05787	HMDB0003325	5280817	1.17	0.07	down	1.11	44.84	up
33.42	166.1174	17-Hydroxyprogesterone	M+2H[2+]	C01176	HMDB0000374	6238	1.21	0.20	down	1.09	5.65	up
33.72	295.2267	13-L-Hydroperoxylinoleic acid	M-H_2O+H[1+]	C04717	HMDB0003871	5280720	1.13	49.25	up	1.06	0.00	down
33.97	147.0601	Naphthalene epoxide	M(S34)+H[1+]	C14786	HMDB0006215	108063	1.06	7.83	up	1.04	0.05	down
33.98	469.3276	Campesterol	M+HCOONa[1+]	C01789	HMDB0002869	173183	1.45	0.17	down	1.38	5.38	up
34.14	980.2760	OPC6-CoA	M-H_4O_2+H[1+]	C16331	HMDB0011114	53480660	1.45	0.03	down	1.23	77.07	up
34.55	140.1069	Epinephrine	M-CO_2+H[1+]	C00788	HMDB0000068	5816	1.36	2.01	up	1.38	0.34	down
34.58	183.1242	Carnosine	M-CO_2+H[1+]	C00386	HMDB0000033	439224	1.17	13.20	up	1.12	0.01	down
34.60	104.1073	Choline	M[1+]	C00114	HMDB0000097	305	1.18	0.22	down	1.34	4.52	up
34.77	110.0091	Thiocysteine	M-CO_2+H[1+]	C01962	HMDB0003585	439614	1.47	2.91	up	1.38	0.35	down
34.96	483.6460	(S)-3-Hydroxydodecanoyl-CoA	M+2H[2+]	C05262	HMDB0003936	440603	1.27	0.06	down	1.21	8.43	up
35.55	139.9636	Phosphoglycolic acid	M-NH_3+H[1+]	C00988	HMDB0000816	529	1.32	2.82	up	1.23	0.38	down
35.62	169.9972	Dihydroxyacetone phosphate	M[1+]	C00111	HMDB0001473	668	1.39	0.48	down	1.29	2.10	up
35.65	172.9957	(S)-Ureidoglycolic acid	M+K[1+]	C00603	HMDB0001005	439269	1.44	0.38	down	1.37	2.85	up
36.66	250.9713	myo-Inositol 1,3,4,5-tetrakisphosphate	M+2H[2+]	C01272	HMDB0001059	107758	1.31	3.38	up	1.26	0.24	down
37.82	139.0391	4-Hydroxybenzoic acid	M+H[1+]	C00156	HMDB0000500	135	1.42	7.96	up	1.27	0.41	down
39.58	114.9675	Methylselenopyruvate	M-HCOONa+H[1+]	C18904	HMDB0060490	53297415	1.25	0.44	down	1.35	3.73	up

Table S2. PPI network of potential anti-inflammatory targets of *Saposhnikoviae Radix*.

BetweennessCentrality	ClosenessCentrality	Degree	Name	BetweennessCentrality	ClosenessCentrality	Degree	Name
0.14783	0.40943	72	TP53	0.00248	0.34972	13	AR
0.08148	0.38612	55	SRC	0.00164	0.32929	13	BRAF
0.06358	0.40372	51	AKT1	0.03093	0.30758	13	RXRA
0.05279	0.39853	47	STAT3	0.00515	0.29604	13	PRKCE
0.04577	0.39029	47	HSP90AA1	0.02126	0.28723	13	CYP1A2
0.02224	0.35429	46	PIK3R1	0.00455	0.32148	12	ICAM1
0.01706	0.36686	44	PIK3CA	0.00645	0.31959	12	TGFB1
0.00956	0.34609	42	PIK3CB	0.01841	0.31935	12	CAMK2B
0.00864	0.34582	41	PIK3CD	0.00197	0.31702	12	CASP8
0.02672	0.38646	40	MAPK1	0.01632	0.31518	12	HSPA5
0.07939	0.38578	39	EP300	0.01311	0.30435	12	MMP9
0.02572	0.38407	39	MAPK3	0.01319	0.34200	10	NR3C1
0.02781	0.37674	39	EGFR	0.00972	0.32854	10	ABL1
0.03201	0.39029	37	ESR1	0.02369	0.31888	10	BAX
0.02060	0.37094	36	HSP90AB1	0.00469	0.31748	10	PLK1
0.07710	0.36227	36	PRKACA	0.00508	0.31472	10	AURKA
0.04011	0.39170	35	JUN	0.00392	0.31156	10	KIT
0.02318	0.36811	32	MAPK8	0.01631	0.30846	10	FGFR1
0.01492	0.36257	32	AKT2	0.00185	0.30824	10	BIRC2
0.01914	0.36076	32	PTK2	0.00864	0.30693	10	PCNA
0.00706	0.33180	32	PTPN11	0.00545	0.30585	10	LCK
0.03242	0.36137	30	TNF	0.00507	0.30435	10	MAP2K7
0.01293	0.35957	29	HRAS	0.00614	0.30307	10	NGFR

0.01232	0.33488	29	PLCG2	0.01252	0.29787	10	GRIA1
0.01286	0.35927	28	MYC	0.00202	0.29767	10	VCAM1
0.02217	0.35897	28	BCL2	0.00342	0.29324	10	MAP2K3
0.02506	0.36563	26	HIF1A	0.00148	0.29324	10	MAP2K6
0.01325	0.36409	26	RELA	0.02916	0.28571	10	DPP4
0.01379	0.36318	25	NFKB1	0.00427	0.28366	10	PPP1CC
0.05373	0.35574	25	FN1	0.00422	0.32904	9	NR4A1
0.01675	0.35400	25	CASP3	0.00928	0.31959	9	NFE2L2
0.01438	0.34748	25	IL6	0.02558	0.31935	9	G6PD
0.00175	0.32461	25	JAK2	0.00481	0.31818	9	NOS3
0.00586	0.35085	24	RAF1	0.00491	0.31564	9	KAT2B
0.00241	0.34748	23	ERBB2	0.01949	0.31472	9	GSTP1
0.00617	0.34119	23	IGF1R	0.00372	0.30435	9	ROCK1
0.00745	0.32412	23	LYN	0.00539	0.30118	9	PSEN1
0.00843	0.32340	23	KDR	0.03771	0.29186	9	PLG
0.01470	0.33774	22	TLR4	0.00235	0.32172	8	EZR
0.02741	0.33565	22	PRKCA	0.01249	0.32148	8	PKM
0.00483	0.36440	21	FOS	0.00505	0.31223	8	BRD4
0.00373	0.34859	21	CCND1	0.00599	0.31044	8	GRIN2B
0.01448	0.34554	21	IKBKB	0.00222	0.30392	8	AURKB
0.00403	0.34335	21	HDAC1	0.01404	0.30181	8	NTRK2
0.00188	0.31633	21	JAK1	0.01202	0.30181	8	MMP2
0.01129	0.34527	20	GSK3B	0.00281	0.30076	8	THRB
0.00315	0.33827	20	MAPK14	0.00533	0.29706	8	IL2
0.00315	0.33827	20	MAPK11	0.00796	0.29206	8	SNCA
0.00189	0.32030	20	PTPN6	0.00261	0.32172	7	HTT

0.00782	0.31679	20	MAP3K7	0.00210	0.31935	7	HDAC6
0.01135	0.34066	19	BCL2L1	0.01595	0.30802	7	PPARA
0.00251	0.34254	18	MDM2	0.01823	0.30097	7	CYP19A1
0.00338	0.31427	18	JAK3	0.00652	0.30076	7	PLA2G4A
0.01554	0.34609	17	HSPA8	0.00583	0.29584	7	LRRK2
0.00802	0.34308	17	ACTB	0.00232	0.29484	7	GRIA2
0.00283	0.34200	17	CDKN1A	0.00476	0.29404	7	VCP
0.03207	0.34093	17	SIRT1	0.00208	0.29384	7	THRA
0.01381	0.32388	17	FGF2	0.01659	0.28515	7	SREBF1
0.00724	0.31610	17	FYN	0.00862	0.32509	6	IGFBP3
0.03021	0.28145	17	ENPP1	0.00890	0.30715	6	TRPV1
0.01344	0.34227	16	MTOR	0.00292	0.30413	6	NOS2
0.01080	0.33827	16	RHOA	0.00137	0.30392	6	YWHAG
0.05160	0.33643	16	PTGS2	0.01566	0.30371	6	PDE3B
0.01056	0.33514	16	MAPT	0.00378	0.29706	6	SIRT2
0.03794	0.33054	16	APP	0.02163	0.28422	6	SLC6A3
0.04940	0.35256	15	GAPDH	0.00815	0.29890	5	SREBF2
0.00149	0.32583	15	PRKCD	0.00287	0.29147	5	PRKAA2
0.01838	0.33906	14	PPARG	0.00146	0.28571	5	ERN1
0.00879	0.32006	14	CXCL8	0.00476	0.28164	5	IRAK4
0.01503	0.30978	14	CCL2	0.00367	0.28091	5	TKT

Table S3. Network diagram of *Saposhnikoviae Radix*-components-targets-inflammation.

BetweennessCentrality	ClosenessCentrality	Degree	Name	BetweennessCentrality	ClosenessCentrality	Degree	Name
0.53589	0.73898	144	Inflammation	0.00174	0.43952	7	SIRT2
0.12214	0.58289	73	Saposhnikoviae Radix	0.00031	0.38313	6	5-O-Methylvisammi nol
0.05172	0.53171	50	PTGS2	0.00065	0.38313	6	N,N-Dicyclohexylur ea
0.03162	0.43513	42	sitosterol	0.00051	0.38313	6	2,4-Dihydroxybenzo phenone
0.02599	0.49772	39	ESR1	0.00048	0.38313	6	panaxynol
0.01953	0.48661	31	DPP4	0.00040	0.38313	6	butylated hydroxytoluene
0.01408	0.41683	30	5-[(Z)-2-(4-hydroxy-3-methoxy-phenyl)vi nyl]resorcinol	0.00121	0.38313	6	ETHYLMYRISTAT E
0.01192	0.41524	28	piceatannol	0.00113	0.43775	6	BCL2L1
0.01044	0.41210	27	2-(3,4-dimethoxyphe nyl)-5-hydroxy-7-me thoxy-chromone	0.00140	0.43775	6	G6PD
0.01491	0.47807	27	ESR2	0.00103	0.43775	6	HSP90AB1
0.01266	0.47598	27	NOS2	0.00091	0.43775	6	IL6
0.01104	0.47391	27	PIK3CA	0.00119	0.43775	6	MAPT
0.01128	0.41210	26	Yangonin	0.00125	0.43775	6	NFKB1
0.00665	0.41210	26	Fraxetin	0.00176	0.43775	6	PDE3B
0.00873	0.41055	25	anomalin	0.00113	0.43775	6	TLR4
0.01151	0.47391	25	KDR	0.00016	0.38179	5	Marmesin

0.01094	0.40901	24	Marmesine	0.00075	0.43600	5	ERN1
0.00746	0.40901	24	Decursin	0.00074	0.43600	5	IGFBP3
0.00651	0.40596	24	(2R,3R)-3-(4-hydroxy-3-methoxy-phenyl)-5-methoxy-2-methylol-2,3-dihydropyrano[5,6-h][1,4]benzodioxin-9-one	0.00073	0.43600	5	LRRK2
0.00746	0.40901	24	Decursin	0.00070	0.43600	5	PTPN11
0.01013	0.47186	24	JAK2	0.00070	0.43600	5	PTPN6
0.00896	0.40596	23	cimifugin	0.00013	0.38045	4	nodakenetin
0.00603	0.40445	23	(2R,3R)-3-(4-hydroxy-3-methoxy-phenyl)-5-methoxy-2-methylol-2,3-dihydropyrano[5,6-h][1,4]benzodioxin-9-one	0.00031	0.38045	4	NSC692928
0.00841	0.40748	23	(3S,8E,10S)-heptadeca-1,8-dien-4,6-diyne-3,10-diol	0.00018	0.38045	4	caryophyllene oxide
0.01576	0.46983	23	AR	0.00037	0.38045	4	Amide HPL
0.00843	0.46983	23	JAK1	0.00009	0.38045	4	11-hydroxy-sec-o-beta-d-glucosylhamaudol_qt
0.00985	0.40596	22	linolenic acid	0.00087	0.43426	4	CASP8
0.00728	0.40596	22	umbelliferone	0.00099	0.43426	4	GAPDH

0.00804	0.46781	22	GSK3B	0.00066	0.43426	4	HIF1A
0.00555	0.40445	21	Scoparone	0.00099	0.43426	4	HSPA8
0.00633	0.40296	21	Linoleic	0.00048	0.43426	4	JUN
0.00546	0.40445	21	7-hydroxy-6,8-dimethoxychromen-2-one	0.00051	0.43426	4	KAT2B
0.01052	0.46581	21	CYP19A1	0.00045	0.43426	4	PKM
0.00838	0.46581	21	CYP1A2	0.00050	0.43426	4	PLA2G4A
0.00802	0.46581	21	HSP90AA1	0.00099	0.43426	4	PRKCE
0.00675	0.40296	20	Guanine	0.00071	0.43426	4	STAT3
0.00776	0.40296	20	Byakangelicol	0.00059	0.43426	4	VCAM1
0.00518	0.40296	20	Ayapanin	0.00004	0.37913	3	Byakangelicin
0.00470	0.40296	20	Uvadex	0.00004	0.37913	3	Nodakenin
0.00576	0.40147	19	psoralen	0.00055	0.43254	3	BAX
0.00611	0.46186	19	AURKA	0.00020	0.43254	3	CAMK2B
0.00585	0.46186	19	SRC	0.00033	0.43254	3	CXCL8
0.00757	0.40000	18	oxybutynin	0.00037	0.43254	3	EP300
0.00511	0.40000	18	linolenate	0.00032	0.43254	3	GRIA2
0.00525	0.40000	18	EIC	0.00025	0.43254	3	GRIN2B
0.00524	0.45992	18	ERBB2	0.00075	0.43254	3	IRAK4
0.00299	0.39854	17	scopoletin	0.00033	0.43254	3	MAP3K5
0.00513	0.39854	17	Norcimifugin	0.00028	0.43254	3	MAP3K7
0.00547	0.45798	17	BRAF	0.00034	0.43254	3	MAPK11
0.00458	0.45417	17	MTOR	0.00020	0.43254	3	MAPK14
0.00523	0.45798	17	PIK3CB	0.00036	0.43254	3	NGFR
0.00473	0.39709	16	L-Tyrosine	0.00043	0.43254	3	NR4A1
0.00366	0.39709	16	Chlorogenic acid	0.00034	0.43254	3	PRKAA2

0.00235	0.39709	16	Phellopterin	0.00025	0.43254	3	PRKACA
0.00434	0.39709	16	Falcarindiol	0.00025	0.43254	3	ROCK1
0.00470	0.39709	16	divaricatacid	0.00038	0.43254	3	THRB
0.00235	0.39709	16	phelloptorin	0.00035	0.43254	3	TKT
0.00235	0.39709	16	Phellopterin	0.00033	0.43254	3	TRPV1
0.00417	0.45607	16	AURKB	0.00025	0.43254	3	VCP
0.00485	0.45607	16	MAPK8	0.00022	0.43254	3	YWHAG
0.00577	0.45607	16	PPARG	0.00107	0.43426	6	
0.00226	0.39564	15	Isobergapten	0.00003	0.37782	2	(2S)-4-methoxy-7-methyl-2-[1-methyl-1-[(2S,3R,4S,5S,6R)-3,4,5-trihydroxy-6-methylol-tetrahydropyran-2-yl]oxy-ethyl]-2,3-dihydrofuro[3,2-g]chromen-5-one
0.00570	0.39564	15	2-(4-METHYL-6-OXO-6H-BENZO(C)CHROMEN-3-YLOXY)-PROPIONIC ACID	0.00001	0.37782	2	Mandenol
0.00287	0.39564	15	Myristicin	0.00003	0.37782	2	5-O-Methylvisammioside
0.00435	0.45417	15	PLK1	0.00015	0.43083	2	ACTB
0.00652	0.45417	15	RXRA	0.00007	0.43083	2	AKT1
0.00181	0.39421	14	Ammidin	0.00019	0.43083	2	AKT2

0.00181	0.39421	14	Ammidin	0.00019	0.43083	2	BIRC2
0.00312	0.39421	14	Prangenidin	0.00007	0.43083	2	CCL2
0.00392	0.45228	14	JAK3	0.00007	0.43083	2	CCND1
0.00539	0.45228	14	MMP9	0.00007	0.43083	2	CDKN1A
0.00401	0.45228	14	PIK3CD	0.00019	0.43083	2	CXCR4
0.00136	0.39279	13	4-hydroxy-9-methoxyfuro[3,2-g]chromen-7-one	0.00016	0.43083	2	ENPP1
0.00504	0.39279	13	Sucrose	0.00012	0.43083	2	EZR
0.00314	0.45041	13	IGF1R	0.00026	0.43083	2	FGF2
0.00560	0.45041	13	NR3C1	0.00007	0.43083	2	FN1
0.00325	0.45041	13	PTK2	0.00026	0.43083	2	FOS
0.00564	0.44856	13	SLC6A3	0.00014	0.43083	2	FYN
0.00161	0.39138	12	divaricatol	0.00014	0.43083	2	GRIA1
0.00356	0.44856	12	BRD4	0.00028	0.43083	2	GSTP1
0.00291	0.44856	12	LYN	0.00053	0.43083	2	HRAS
0.00393	0.44856	12	MMP2	0.00015	0.43083	2	HTT
0.00201	0.38998	11	isopimpinellin	0.00026	0.43083	2	IL2
0.00189	0.38998	11	Ostenol	0.00014	0.43083	2	MAP2K3
0.00243	0.44672	11	ABL1	0.00019	0.43083	2	MAP2K6
0.00375	0.44672	11	CASP3	0.00015	0.43083	2	MYC
0.00227	0.44490	10	APP	0.00010	0.43083	2	NFE2L2
0.00331	0.44490	10	HDAC1	0.00007	0.43083	2	NTRK2
0.00268	0.44490	10	PRKCD	0.00015	0.43083	2	PCNA
0.00060	0.38721	9	ledebouriellol	0.00016	0.43083	2	PLCG2
0.00262	0.44309	9	BCL2	0.00011	0.43083	2	PLG

0.00288	0.44309	9	HDAC6	0.00053	0.43083	2	PPP1CC
0.00199	0.44309	9	KIT	0.00012	0.43083	2	PSEN1
0.00226	0.44130	9	MAP2K7	0.00022	0.43083	2	RHOA
0.00209	0.44309	9	RELA	0.00012	0.43083	2	SIRT1
0.00075	0.38584	8	Majudin	0.00014	0.43083	2	SNCA
0.00062	0.38584	8	isoimperatorin	0.00026	0.43083	2	SREBF1
0.00218	0.38584	8	beta-sitosterol	0.00026	0.43083	2	SREBF2
0.00146	0.44130	8	ICAM1	0.00040	0.43083	2	TGFB1
0.00160	0.44130	8	IKBKB	0.00022	0.43083	2	THRA
0.00280	0.44130	8	MDM2	0.00007	0.43083	2	TNF
0.00163	0.44130	8	PPARA	0.00007	0.43083	2	TP53
0.00139	0.44130	8	RAF1	0.00000	0.36887	1	methyl icosa-11,14-dienoate
0.00180	0.38448	7	Sec-O-Glucosylhamaudol	0.00000	0.42578	1	EGFR
0.00141	0.43952	7	FGFR1	0.00000	0.42578	1	HSPA5
0.00119	0.43952	7	LCK	0.00000	0.42578	1	MAPK1
0.00133	0.43952	7	MAPK3	0.00000	0.42578	1	NOS1
0.00107	0.43952	7	PIK3R1	0.00000	0.42578	1	NOS3
0.00229	0.43952	7	PRKCA				

Table S4. The pathways - targets - components of *Saposhnikoviae Radix* in the treatment of rheumatoid arthritis.

Pathways	Targets	Components
AGE-RAGE signaling pathway in diabetic complications	PIK3R1, AKT1, STAT3	Sitosterol
		5-O-methylvisammioside
	PIK3R1, AKT1, HSP90AA1, TP53	Hamaudol
		Sec-O-Glucosylhamaudol
		5-O-methylvisammioside
PI3K-Akt signaling pathway		6-Sitosterol
	PIK3R1, AKT1	Sitosterol
		Sec-O-Glucosylhamaudol
TNF signaling pathway		5-O-methylvisammioside
	AKT1,TP53	Sec-O-Glucosylhamaudol
MAPK signaling pathway		5-O-methylvisammioside
	PIK3R1, AKT1	Sitosterol
		Sec-O-Glucosylhamaudol
Toll-like receptor signaling pathway		5-O-methylvisammioside
